# Prevalence and Antimicrobial Resistance of *Staphylococcus aureus* and Coagulase-Negative *Staphylococcus*/*Mammaliicoccus* from Retail Ground Meat: Identification of Broad Genetic Diversity in Fosfomycin Resistance Gene *fosB*

**DOI:** 10.3390/pathogens11040469

**Published:** 2022-04-14

**Authors:** Masako Osada, Meiji Soe Aung, Noriko Urushibara, Mitsuyo Kawaguchiya, Nobuhide Ohashi, Mina Hirose, Nobumichi Kobayashi

**Affiliations:** 1 Master’s Program in Midwifery, Tenshi College Graduate School, Sapporo 065-0013, Japan; osada.masako@tenshi.ac.jp; 2 Department of Hygiene, Sapporo Medical University School of Medicine, Sapporo 060-8556, Japan; noriko-u@sapmed.ac.jp (N.U.); kawaguchiya@sapmed.ac.jp (M.K.); ohashin@sapmed.ac.jp (N.O.); nkobayas@sapmed.ac.jp (N.K.); 3 Division of Pediatric Dentistry, Department of Oral Growth and Development, School of Dentistry, Health Sciences University of Hokkaido, Ishikari-Tobetsu 061-0293, Japan; minaniwa@hoku-iryo-u.ac.jp

**Keywords:** retail meat, *Staphylococcus aureus*, coagulase-negative *Staphylococcus*, multilocus sequence typing, *fosB*

## Abstract

*Staphylococcus* is a major bacterial species that contaminates retail meat products. The objective of this study was to clarify the prevalence, antimicrobial resistance and genetic determinants of *Staphylococcus*/*Mammaliicoccus* species in retail ground meat in Japan. From a total of 146 retail ground meat samples (chicken, pork, mixed beef/pork) purchased during a 5-month period, 10 *S. aureus* and 112 isolates of coagulase-negative staphylococcus (CoNS)/*Mammaliicoccus* comprising 20 species were recovered. *S. aureus* isolates were classified into five genetic types, i.e., *coa*-IIa/ST5, *coa*-VIc/ST352 (CC97), *coa*-VIIb/ST398, *coa*-Xa/ST15, and *coa*-XIc/ST9, which were all related to those of livestock-associated clones. All the staphylococcal isolates were *mecA*-negative and mostly susceptible to all the antimicrobials tested, except for ampicillin among *S. aureus* (resistance proportion; 50%). Among CoNS, the fosfomycin resistance gene *fosB* was prevalent (30/112; 26.8%), primarily in *S. capitis*, *S. warneri*, and *S. saprophyticus*. Phylogenetic analysis of *fosB* revealed the presence of seven clusters, showing broad diversity with 65–81% identity among different clusters. In the CoNS isolates from ground meat samples, *fosB* was assigned into three clusters, and *S. saprophyticus* harbored the most divergent *fosB* with three genetic groups. These findings suggested the circulation of multiple *fosB*-carrying plasmids among some CoNS species.

## 1. Introduction

*Staphylococcus* is a common commensal bacteria that inhabits skin and mucous membranes of various parts of the body in humans and animals [1]. This genus is recognized as a major pathogenic microorganism and causes a wide spectrum of diseases. The genus *Staphylococcus* consists of at least 62 species (https://lpsn.dsmz.de/genus/staphylococcus; accessed on 1 March 2022) as of March 2022, which have been classified into coagulase-positive and -negative staphylococcus (CoPS and CoNS, respectively), and coagulase-positive/variable staphylococcus [2]. CoPS includes major pathogenic species, i.e., *Staphylococcus aureus*, and three other species (*S. argenteus*, *S. schweitzeri*, and *S. singaporensis*) that form *S. aureus* complex [3,4], with *S. argenteus* being increasingly reported as a human pathogen worldwide [5]. Coagulase-positive/variable staphylococcus consists of several species represented by *S. hyicus* and *S. intermedius* [2]. Although CoNS colonizes healthy individuals more rigidly than *S. aureus* and is thus considered less virulent, some species/strains of CoNS are recognized as causes of specific infections (e.g., device-related infections), associated with increased drug resistance and biofilm formation [2,6]. Recently, five former CoNS species (*S. sciuri* group) were reclassified into the genus *Mammaliicoccus* (e.g., *M. sciuri*) [7].

Staphylococci originating from animals harbor a wide variety of antimicrobial resistance (AMR) genes [8]. Part of the AMR genes shared by humans and animals (e.g., *tet*(L), *cfr*, *fexA*, and *dfrK*) have already been identified and more commonly in animal-related staphylococci, suggesting an animal origin. Methicillin-resistant *S. aureus* (MRSA), one of the most important antimicrobial-resistant bacteria, carries a composite SCC*mec* element containing *mecA*, a determinant of methicillin-resistance. The origin of *mecA* was also presumed to be an animal-related species, *Staphylococcus fleurettii* (*Mammaliicoccus fleurettii*) [9], which is distributed to pigs, cows and other animal products [10]. Meat products, as well as dairy products are commonly contaminated with *S. aureus* and CoNS [11,12]. A *mecA* homologue, *mecC* is also distributed to humans and animals at low prevalence [8]. Those foods are considered a potential vehicle for the transmission of staphylococcus, mediating the introduction of AMR genes and/or virulence factor genes to the human population.

To date, numerous published reports have described the prevalence of *S. aureus*/MRSA isolated from retail meat products in many countries around the world, revealing their AMR traits and genotypes [13,14,15,16,17,18,19,20,21,22]. Though much less information is available, increased AMR rates in various CoNS species from meat have also been shown in some studies [10,11,23]. In Japan, information on *S. aureus* in retail meat is limited to some studies for older isolates [24,25,26], while there is no data on CoNS. Therefore, we conducted this study to reveal the prevalence of *Staphylococcus* and *Mammaliicoccus* in retail ground meat in Japan, their AMR and its responsible genetic determinants.

Fosfomycin is a broad-spectrum bactericidal antibiotic that interferes with cell wall biosynthesis, via inhibition of the MurA enzyme catalizing peptidoglycan precursor, which is a different mechanism from that of beta-lactams [27]. Resistance to fosfomycin through the fosfomycin-inactivating enzyme (FosB) has been occurring in *S. aureus*/MRSA clinical isolates, posing concern for treatment [28,29,30]. Though prevalence of fosfomycin resistance in staphylococci from meat has been scarcely studied, we revealed relatively high prevalence of fosfomycin resistance gene *fosB* in CoNS. Through the phylogenetic analysis of the broad genetic diversity of *fosB*, we proposed a reclassification scheme of *fosB* genetic groups of staphylococcal species.

## 2. Results

### 2.1. Isolation of Staphylococcus/Mammaliicoccus Isolates

A total of 146 packages of retail ground meat products were purchased from several grocery stores located in Sapporo and its neighboring towns in the Hokkaido prefecture, in the northern main island of Japan, during a 5-month period (from May to September 2021). These meat products were collected by convenience sampling, and comprised chicken (*n* = 93), pork (*n* = 22), and a mixture of beef and pork (*n* = 31). All the samples were non-frozen raw meat and were kept at a low temperature (<10 ℃) in the retail outlet. Purchased samples were placed in a portable cold insulation bag and transported to the laboratory.

From the 146 ground meat specimens, 10 *S aureus* isolates (6.8%) and 112 isolates of CoNS/*Mammaliicoccus* were recovered (Table 1). The proportion of *S. aureus* from the mixed ground meat (beef and pork) (16%) was higher than that from chicken and pork. CoNS/*Mammaliicoccus* consisted of 20 species (16 *Staphylococcus* and 4 *Mammaliicoccus* species), with *S. saprophyticus* being the most common, followed by *M. sciuri*, *S. warneri*, *S. pasteuri*, *S. capitis*, and *S. chromogenes*. From the three types of ground meat, *S. saprophyticus* was commonly isolated with a similar prevalence rate (15–18%), as well as *S. pasteuri* (5–9%).

### 2.2. AMR and Genetic Characterization of S. aureus

The 10 *S. aureus* isolates were all *mecA*-negative (methicillin-susceptible) and genetically classified into five genetic groups (coagulase genotype/sequence type (clonal complex)), i.e., *coa*-IIa/ST5, *coa*-VIc/ST352 (CC97), *coa*-VIIb/ST398, *coa*-Xa/ST15, and *coa*-XIc/ST9 (CC1) (Table 2). ST15 and ST352 *S. aureus* were susceptible to all the antimicrobials tested, and other isolates showed resistance to a few antimicrobials (ampicillin, erythromycin, clindamycin, and levofloxacin), harboring *blaZ* or *erm*(C). Resistance proportion (percentage) to ampicillin of *S. aureus* (50%) was significantly higher than CoNS/*Mammaliicoccus* (19.6%) (Table 3). None of the isolates had PVL genes and ACME-*arcA*. An enterotoxin gene *seb* was detected in ST15 isolates, while the enterotoxin gene cluster (*egc*) (*seg-sei-sem-sen-seo*) was detected in ST5 and ST9 isolates. A leukocidin gene *lukM* was identified only in an isolate of ST352.

### 2.3. AMR and Antimicrobial Resistance Genes in CoNS/Mammaliicoccus

Resistance proportions to individual antimicrobials and prevalence of resistance genes in each CoNS/*Mammaliicoccus* species are shown in Table 3. Distribution of MIC to eight antimicrobials was illustrated in Figure S1. CoNS/*Mammaliicoccus* derived from meat samples were susceptible to most of the antimicrobials, while 8–20% of the isolates showed resistance to ampicillin, gentamicin, clindamycin, and tetracycline. For antimicrobial susceptibility testing, we employed a commercial kit (Dry Plate Eiken DP32, Eiken Chemical, Tokyo, Japan) based on the broth microdilution method to test 18 antimicrobials, including fosfomycin. However, the agar dilution method is recommended for susceptibility testing of fosfomycin [31]. Accordingly, results of fosfomycin are not shown in Table 1, but mentioned here as reference information; 51 CoNS/*Mammaliicoccus* isolates (45.5%) showed an MIC of ≥64 μg/mL (46 isolates, ≥128 μg/mL), representing presumptive resistance to fosfomycin, while all the *S. aureus* isolates were susceptible to fosfomycin.

Among the CoNS species with multiple isolates, *S. warneri* showed the highest resistance proportions to ampicillin and gentamicin, harboring *blaZ* and aminoglycoside modifying enzyme (AME) genes (*aac(6′)-Ie-aph(2”)-Ia*, *ant(4′)-Ia*). Resistance to erythromycin and clindamycin was the most common in *S. chromogenes*, associated with *erm*(C) and *lnu*(B), despite the low number of isolates (*n* = 6). Tetracycline resistance was found in 10 species, with *tet*(K) being the most common, followed by *tet*(M). The fosfomycin resistance gene, *fosB* was found to be prevalent among CoNS/*Mammaliicoccus* (30 isolates; 26.8%). High prevalence of *fosB* was noted in *S. capitis* (63%, 5/8), *S. warneri* (62%, 9/14), and *S. saprophyticus* (57%, 13/23), while *S. lugdunensis* and *S. pasteuri* also harbored this gene. All the isolates were negative for *optrA*, *fexA*, and *cfr*, showing susceptibility to linezolid (MIC, 0–2 μg/mL).

### 2.4. Phylogenetic Analysis of fosB

**Figure 1:**
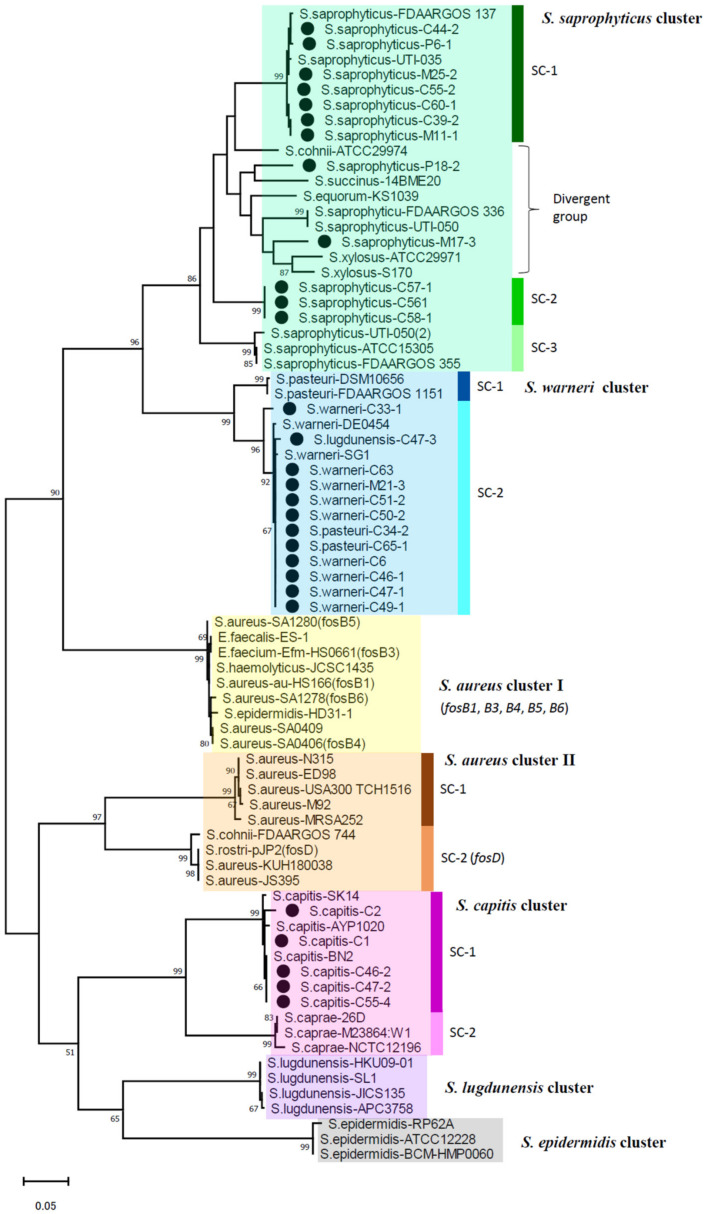
Phylogenetic dendrogram of *fosB* belonging to FosB-S clade constructed by maximum-likelihood method with MEGA.11 program. Trees were statistically supported by bootstrapping with 1000 replicates, and genetic distances were calculated by the Kimura two-parameter model. Variation scale is described at the bottom. Percent bootstrap support is indicated by the values at each node (the values <80 are omitted). Filled circles indicate staphylococcal isolates analyzed in the present study, and sequences of other strains were retrieved from the GenBank database. Seven clusters are shown on the right. Strains included in these clusters are drawn with different colors. Subclusters (SC) are also indicated on the right with vertical bars.

The nucleotide sequence of *fosB* was determined for most of the *fosB*-positive isolates (*n* = 29) and was phylogenetically analyzed with representative *fosB* sequences available in the GenBank database, which were grouped as a *Staphylococcus* cluster (FosB-S) by Song et al. [32]. The constructed phylogenetic tree of *fosB* (Figure 1) revealed the presence of at least seven clusters discriminated by high bootstrap values at the nodes of branches (>85). Because the dominant staphylococcus species were evident in individual clusters, these species names were assigned to the designation of clusters. Nevertheless, two distinct clusters were revealed for *S. aureus* (I and II), and subclusters (SC) were differentiated for four clusters. The *fosB* genes identified in the present study were assigned to three clusters: a *S. saprophyticus* cluster, *S. warneri* cluster, and *S. capitis* cluster. *fosB* of *S. saprophyticus* in this study was classified into three distinct groups within a cluster (*S. saprophyticus* cluster SC-1, SC-2, and divergent group). *fosB* of *S. lugdunensis* and *S. pasteuri* were grouped into the *S. warneri* cluster.

As reported for FosB-S [29,32], *fosB* of *S. saprophyticus* and *S. warneri* clusters in the present isolates comprised 420 nucleotides encoding a 139-amino acid protein. However, FosB of all the isolates of *S. capitis* cluster in the present study was one-amino acid longer (i.e., 140 amino acids), which was also found in the reference sequence of *S. capitis* in GenBank (Figure S2). The nucleotide sequence identity of *fosB* among the different clusters were analyzed for those of the present isolates together with those available in the GenBank database (Table S1). *fosB* was revealed to be highly divergent, showing a 65–81% identity among different clusters, with a 79–100% identity within the same cluster. In particular, clusters of *S. saprophyticus*, *S. aureus*-II, and *S. capitis* exhibited more diversity than other clusters. While *fosB* sequences of *S. saprophyticus* exhibited 86–99% identity within the cluster, *fosB* of two isolates M17-3 and P18-2 showed 87–92% identity to those of other *S. saprophyticus* isolates in the present study, as well as any *fosB* sequences in the GenBank database. In contrast, within the *S. warneri* cluster (SC-2) and the *S. capitis* cluster (SC-1), the nucleotide sequence identity of *fosB* was more than 97% (Table S1, Figure S2).

### 2.5. Prevalence of 6-TG Synthesis Genes among CoNS/Mammaliicoccus

Recently, some CoNS species were reported to produce 6-thioguanine (6-TG), which suppresses the growth of *S. aureus* [33]. To examine the possible association of 6-TG synthesis in CoNS and the isolation of *S. aureus*, the presence of three genes (*tgsB*, *tgsC*, *tgsD*) included in the 6-TG biosynthetic gene cluster was analyzed by PCR using newly designed primers (Table S2). The 6-TG synthesis genes were detected in only *S. capitis* (3 isolates) and *S. chromogenes* (2 isolates) among all the CoNS/*Mammaliicoccus* isolates (Table 4). Nevertheless, only two genes (*tgsC* and *tgsD*) were found in *S. chromogenes*. From the meat samples with *tgs*-positive *S. capitis* or *S. chromogenes*, *S. aureus* was not isolated, while other staphylococcal species were recovered. Among the 10 *S. aureus* isolates, six isolates with ST9, ST15, and ST398 were isolated with other staphylococcal species from the same meat specimens, including *S. chromogenes*, *S. warneri*, *M. sciuri* (Table 5). Two *S. chromogenes* isolates co-isolated with *S. aureus* were negative for the *tgs* genes.

## 3. Discussion

Prevalence of *S. aureus*/MRSA contaminating retail meat has been reported worldwide, while their isolation proportions vary by individual studies. In several recent studies in Asia and the Middle East, the isolation frequency of *S. aureus* from raw meat ranged from about 10 to 21% [11,17,18,22,34], while the higher prevalence of *S. aureus* (>28%) with the detection of MRSA (generally ~8% of *S. aureus*) was described in the US, Africa, and China [14,16,21,35,36,37]. Although information in Japan is available in only a few old studies (2002–2006), the isolation frequency of *S. aureus* was 66% in raw chicken meat [24], and 33% in retail raw meat (3% of MRSA), with a higher prevalence in chicken than pork/beef [25]. In contrast, in the present study, *S. aureus* was isolated at a lower level (6.8%) compared with those in the above-mentioned reports, without the detection of MRSA, showing a higher proportion of *S. aureus* in mixed beef/pork than in chicken. Such difference in the prevalence of *S. aureus*/MRSA may be caused by the study design, including the sample number and period, culture method, and environmental conditions at the study site.

Genotypes of *S. aureus* isolates in the present study were *coa*-IIa/ST5, *coa*-XIc/ST9, *coa*-VIc/ST352 (CC97), *coa*-VIIb/ST398, and *coa*-Xa/ST15, among which ST398 (CC398) has been the most frequently reported for *S. aureus*/MRSA from retail meat, particularly pork [15,19,20,35,38,39], and is known as that of livestock-associated *S. aureus* [40]. Other STs, i.e., ST5, ST9, ST15, and CC97, were also reported for animal-associated types [41,42] and isolates from meat samples, with ST5 and ST9 being more common than ST398 occasionally [14,19,20,35,39,43]. The gene of a leukocidin *lukM*, which is involved in bovine mastitis [44] and is scarcely detected in human clinical isolates, was identified in an ST352 (CC97) isolate in the present study, which may also suggest its relation to animals.

While CoNS/*Mammaliicoccus* isolates in the present study were susceptible to most of the antimicrobials, a higher resistance proportion to some drugs (ampicillin, gentamicin, clindamycin, tetracycline, fosfomycin) were noted for *S. chromogenes*, *S. pasteuri*, *S. saprophyticus*, and *S. warneri*, associated with resistance genes *blaZ*, *erm*(C), *aac(6′)-Ie-aph(2″)-Ia*, *tet*(K), *tet*(M), and *fosB*. In particular, *S. warneri* showed multiple drug resistance. Although much less work has been done on the drug resistance of CoNS from meat products, *S. chromogenes*, *S. epidermidis*, *S. saprophyticus*, and *S. xylosus* were described as common species showing resistance to tetracycline harboring *tet*(K), *tet*(L), or *tet*(M) [23,45,46].

In the present CoNS study, *fosB* was identified in 60% of the presumptive fosfomycin-resistant isolates (30/51). This incidence of *fosB* may be comparable to that reported for *S. aureus* from milk samples (67% of fosfomycin-resistant isolates) [47]. Except for *fosB*, mutations in *murA*, *uhpT*, and *glpT* were revealed as the mechanism of fosfomycin resistance in *Staphylococus* [30], which are suggested to be responsible for the fosfomycin resistance in *fosB*-negative isolates. Nevertheless, these mutations were commonly identified in hospital-associated, fosfomycin-resistant *S. aureus*, despite low prevalence of *fosB* [48]. Thus, *fosB*-associated resistance appears to be more related to CoNS distributed to animals. Although *fosB* in *Staphylococci* from calves and dogs was detected in a few reports [49,50], its prevalence in individual animal species has been scarcely understood. Thus, *fosB*-carrying bacteria in animals will be of significance to be studied in the future. Furthermore, because a high proportion of fosfomycin resistance was described for *S. saprophyticus* from urogenital infections [51], this resistance in CoNS should be carefully monitored for clinical isolates.

FosB is Mg^2+^ dependent thioltransferase encoded by *fosB* located in plasmid, one of the four clades (*fosA*, *fosB*, *fosC*, and *fosX*) [28]. *fosB* is distributed to Gram-positive bacteria and is phylogenetically differentiated into three groups, fosB-B1 and fosB-B2 in *Bacillus*, and *fosB-S* in *Staphylococcus* [32]. In the present study, we revealed broad genetic diversity among *fosB-S* genes, including those identified in the isolates from meat, and the presence of distinct clusters related to staphylococcal species. Remarkably, *fosB* in *S. saprophyticus* was the most divergent, including at least three genetic groups in our isolates, suggesting the circulation of plasmids harboring different *fosB* genes in this species. Through the phylogenetic analysis, the previously described designation of staphylococcal *fosB* could be reassigned to the clusters revealed in the present study: “*fosB1*, *fosB3*–*fosB6*” described for MRSA [29] were classified into the *S. aureus* cluster I, *fosD* into *S. aureus*, *S. rostri*, and *S. arlettae* [52,53,54] was grouped into the *S. aureus* cluster II-SC2 (Figure 1).

Production of 6-TG is a newly identified anti-*S. aureus* mechanism of some CoNS species [33], unlike the already known antimicrobial peptide, i.e., bacteriocin [55]. In our present study, the presence of the 6-TG biosynthetic gene (*tgs*) cluster was detected in *S. capitis* and *S. chromogenes* isolates, which supported the finding through the survey of *tgs* operon among genomic data [33]. Although *S. aureus* was not co-isolated from the samples with *tgs*-positive CoNS in our study, the inhibition effect of 6-TG in the natural environment is still not clear, because of low numbers of *S. aureus* and *tgs*-positive isolates. Further epidemiological study is necessary to evaluate the effect of 6-TG from CoNS to the prevalence of *S. aureus* in nature.

In the present study, we revealed the low prevalence of *S. aureus* and the species diversity of CoNS/*Mammaliicoccus* in ground meat products in Japan, along with the prevalence of CoNS with divergent *fosB*. These observations indicated the need for periodic surveillance of staphylococci in raw meat products to reveal change in the ecological nature of bacteria, which may be potentially affected by the practice of the livestock industry.

## 4. Materials and Methods

### 4.1. Isolation and Identification of Staphylococcus/Mammaliicoccus Species

A 10-g portion of ground meat sample was aseptically taken and transferred into a sterile plastic tube containing 5 mL of Mueller Hinton broth (Becton, Dickinson and Company, Sparks, MD, USA), followed by stirring with a vortex mixer and subsequent enrichment culture at 37 °C for 5 h. Thereafter, a loopful of the culture was streaked on CHROMagar™ Staph aureus (Kanto Chemical, Tokyo, Japan) agar plates, followed by incubation at 37 °C for 48 h aerobically. *Staphylococcus*-like colonies grown on the agar plates were picked up and subcultured on blood agar plates by incubation at 37 °C overnight. Bacterial species in the isolates were identified genetically by a determination of the partial sequence of the 16S rRNA gene (approx. 1500-bp) as reported previously [56]. For species identification, >99% identity of the 16S rRNA sequence revealed by BLAST^R^ search (https://blast.ncbi.nlm.nih.gov/Blast.cgi, accessed on 1 December 2021) was employed. The presence of *nuc*, *mecA*, Panton–Valentine leucocidin (PVL) and arginine catabolic element (ACME)-*arcA* genes was examined for all the isolates by multiplex PCR as described by Zhang et al. [57]. For all the isolates assigned to *S. aurues*, a PCR targeting non-ribosomal peptide synthetase (*nrps*) gene was performed to discriminate from *S. argenteus* [58]. From a single meat sample, multiple isolates showing different colonial morphology on CHROMagar plates were picked up and analyzed. However, only one isolate representing a single *Staphylococccus*/*Mammaliicoccus* species was selected for further characterization.

### 4.2. Antimicrobial Susceptibility Test

For all the isolates, antimicrobial susceptibility was measured by broth microdilution test using a Dry Plate Eiken DP32 (Eiken Chemical, Tokyo, Japan). Minimal inhibitory concentrations (MICs) within limited ranges were measured for 18 antimicrobial agents: beta-lactam (oxacillin(OXA), cefoxitin (FOX), ampicillin (AMP), cefazoline (CFZ), cefmetazole (CMZ), flomoxef (FMX), imipenem (IPM)), aminoglycoside (gentamicin(GEN), arbekacin (ABK)), macrolide (erythromycin (ERY)), lincosamide (clindamycin (CLI)), glycopeptide (vancomycin (VAN), teicoplanin (TEC)), fluoroquuinolone (levofloxacin (LVX)), tetracycline (minocycline (MIN)), and others (linezolid (LZD), fosfomycin (FOF), and trimethoprim/sulfamethoxazole (SXT)), and resistance was judged according to breakpoints mentioned in the Clinical Laboratory Standards Institute (CLSI) standards (2018) [59] except for FOF, ABK and FMX. We referred to the European Committee on Antimicrobial Susceptibility Testing (EUCAST) breakpoint for FOF (Resistance, MIC of >32 µg/mL, *Staphylococcus* spp.) measured in agar dilution method [60], and employed a unique breakpoint for ABK (4 µg/mL, which is higher than the 2 µg/mL defined by the Japanese Society of Chemotherapy for a respiratory infection), and a breakpoint of FMX (16 µg/mL) defined by the Japanese Society of Chemotherapy for a urinary tract infection [61]. MIC of Tetracycline (TET) was measured manually using a broth microdilution test for all the isolates

### 4.3. Detection of Antimicrobial Resistance Genes, Genetic Analysis of fosB

Presence of genes conferring resistance to penicillin (*blaZ*), macrolides-lincosamides-streptogramins (*erm*(A), *erm*(B), *erm*(C), *msrA*, *lnuA*, *lnuB*), aminoglycosides (*aac(6′)-Im*, *aac(6′)-Ie-aph(2″)-Ia*, *ant(3″)-Ia*, *ant(4′)-Ia*, *ant(6)-Ia*, *ant(9)-Ia*, *ant(9)-Ib*, *aph(2″)-Ib*, *aph(2″)-Ic*, *aph(2″)-Id* and *aph(3′)-IIIa*), oxazolidinone, phenicols, lincosamide, and pleuromutilins (*optrA*, *cfr*) were examined by a uniplex or multiplex PCR using the primers previously reported [62,63]. For the detection of *fosB*, primers for PCR detection were newly designed in this study depending on each staphylococcal species (Table S3), based on sequence information in the GenBank database, because *fosB* is genetically divergent. All the primer pairs were attempted to detect *fosB*. The full-length *fosB* gene sequence was determined by Sanger sequencing for the PCR products with primers shown in Table S4, using a BigDye Terminator v3.1 Cycle Sequencing kit (Applied Biosystems, Foster City, CA, USA) on an automated DNA sequencer (ABI PRISM 3100, Applied Biosystems, Foster City, CA, USA). A phylogenetic dendrogram of *fosB* was constructed by the maximum likelihood method using the MEGA11 software, together with *fosB* sequence data of staphylococcal strains available in the GenBank database. Multiple alignments of *fosB*/FosB and calculation of sequence identity were performed using the Clustal Omega program (https://www.ebi.ac.uk/Tools/msa/clustalo/, accessed on 1 December 2021). Sequence data of *fosB* determined in the present study were deposited in the GenBank database under accession numbers shown in Table S5. PCR detection of *tgsB*, *tgsC*, *tgsD* genes in the 6-TG biosynthetic gene cluster was performed for all the CoNS/*Mammaliicoccus* using the primers listed in Table S2.

### 4.4. Genotyping and Detection of Virulence Factors

For *S. aureus* isolates, the genotype of the staphylocoagulase gene (*coa*) was determined through sequencing of the partial *coa* gene (D1, D2 and the central regions) as described previously [56]. The sequence type (ST) based on a multilocus sequencing typing (MLST) scheme [64] and *spa* type based on protein A gene X-region [65] were determined. The presence of gene encoding following virulence factors in *S. aureus* was analyzed by multiplex or uniplex PCRs as described previously [62,66]: staphylococcal enterotoxin (SE) (-like) genes (*sea-see*, *seg-seu*, *selw*, *selx*, *sey*, *selz*, *sel26*, *sel27*), hemolysins (*hla*, *hlb*, *hld*, *hlg*), leukocidines (*lukDE*, *lukM*, *LukS-PV-lukF-PV*), toxic-shock syndrome toxin-1 (*tst-1*), adhesins (*clfA*, *clfB*, *cna*, *ebpS*, *eno*, *fib*, *fnbA*, *fnbB*, *icaA*, *icaD*, *sdrC*, *sdrD*, *sdrE*), immune evasion factors (*chp*, *sak*, *scn*).

### 4.5. Statistical Analysis

Statistical analyses were performed by IBM SPSS Statistics ver.26. The Chi-square test was used to analyze the differences in the isolation rate of *S. aureus* and the proportion of AMR/drug resistance genes depending on the staphylococccal species. A *p*-value < 0.05 was considered statistically significant.

## Figures and Tables

**Table 1:** Isolation of *S. aureus*, CoNS/*Mammaliicoccus* species from different types of ground meat specimens.

Type of Ground Meat (No. of Samples)	No. of *S. aureus* Isolates (% in Each Type of Ground Meat)	CoNS *^1^/*Mammaliicoccus* Species	
		No. of Isolates	Bacterial Species (No. of Isolates)
Chicken (93)	3 (3.2%)	61	*S. agnetis* (1), *S. capitis* (8), *S. carnosus* (2), *S. chromogenes* (3), *S. cohnii* (5), *S. condimenti* (3), *S. lugdunensis* (1), *S. pasteuri* (8), *S. saprophyticus* (14), *S. warneri* (8), *S. xylosus* (1), *M. sciuri* (4), *M. stepanovicii* (3)
Pork (22)	2 (9.1%)	16	*S. agnetis* (1), *S. hyicus* (1), *S. pasteuri* (1), *S. rostri* (1), *S. saprophyticus* (4), *S. warneri* (1), *M. sciuri* (6), *M. vitulinus* (1)
Beef and pork (31)	5 (16.1%) *^2^	35	*S. chromogenes* (3), *S. haemolyticus* (3), *S. kloosii* (1), *S. pasteuri* (2), *S. rostri* (1), *S. saprophyticus* (5), *S. sciuri* (10), *S. simulans* (1), *S. warneri* (5), *M. lentus* (1), *M. vitulinus* (3)
Total (146)	10 (6.8%)	112	*S. saprophyticus* (23), *M. sciuri* (20), *S. warneri* (14), *S. pasteuri* (11), *S. capitis* (8), *S. chromogenes* (6), *S. cohnii* (5), other 13 species (25)

*^1^ An isolate of *S. hyicus* was assigned as CoNS because this isolate was confirmed to be coagulase gene-negative by PCR. *^2^ *p* < 0.05.

**Table 2:** Genotypes, antimicrobial resistance profile, resistance genes and virulence factors in 10 *S. aureus* isolates from retail ground meat specimens.

Isolate ID *^1^	Specimen Type	*coa* Type	ST (CC) *^2^	*spa* Type	Resistance Profile *^3^	Resistance Gene *^4^	Virulence Factor (Gene) Profile *^5^
C26	Chicken	IIa	ST5 (CC5)	t3478	All susceptible	ND	*lukDE*, *hla*, *hlb*, *hld*, *hlg*, *seg*, *sei*, *sem*, *sen*, *seo*, *selx*, *selw*, *eno*, *fib*, *sdrD*
C35	Chicken	IIa	ST5 (CC5)	t3478	All susceptible	ND	*lukDE*, *hla*, *hlb*, *hld*, *hlg*, *seg*, *sei*, *sem*, *sen*, *seo*, *selx*, *selw*, *eno*, *fib*, *sdrD*
M3	Pork and beef (mixed)	VIc	ST352 (CC97)	t5695	All susceptible	ND	*lukDE*, *lukM*, *hla*, *hlb*, *hld*, *hlg*, *selx*, *selw*, *fib*, *sdrD*
M4-2	Pork and beef (mixed)	VIIb	ST398	t571	AMP, LVX	*blaZ*	*hla*, *hlb*, *hld*, *hlg*, *eno*
P12-1	Pork	VIIb	ST398	t1419	ERY, CLI-i	*erm(C)*	*hla*, *hlb*, *hld*, *hlg*, *eno*
M22-1	Pork and beef (mixed)	VIIb	ST398	t1419	ERY, CLI-i	*erm(C)*	*hla*, *hlb*, *hld*, *hlg*, *eno*
C25-1	Chicken	Xa	ST15	t084	AMP	*blaZ*	*lukDE*, *hla*, *hlb*, *hld*, *hlg*, *seb*, *selx*, *selw*, *eno*, *fib*, *sdrD*, *scn*
M20-1	Pork and beef (mixed)	Xa	ST15	t5819	AMP	*blaZ*	*lukDE*, *hla*, *hlb*, *hld*, *hlg*, *seb*, *selx*, *selw*, *eno*, *fib*, *sdrD*, *scn*
M2	Pork and beef (mixed)	XIc	ST9 (CC1)	t337	AMP	*blaZ*	*hla*, *hlb*, *hld*, *seg*, *sei*, *sem*, *sen*, *seo*, *selx*, *selw*, *sey*, *sel26*, *sel27*, *eno*, *fib*, *sdrD*
P7-1	Pork	XIc	ST9 (CC1)	t337	AMP	*blaZ*	*hla*, *hlb*, *hld*, *seg*, *sei*, *sem*, *sen*, *seo*, *selx*, *selw*, *sey*, *sel26*, *sel27*, *eno*, *fib*, *sdrD*

*^1^ Code number of isolate. *^2^ ST, sequence type; CC, clonal complex. *^3^ AMP, ampicillin; LVX, levofloxacin; ERY, erythromycin. CLI-i, inducible resistance to clindamycin. *^4^ ND, not detected. *^5^ All the isolates had the following adhesin genes: *ebpS*, *fnbA*, *fnbB*, *icaA*, *icaD*, *clfA*, *clfB*, *sdrC*, and *sdrE*. All the isolates were negative for *sea*, *sec*, *sed*, *see*, *sek*, *seq*, *ses*, *set*, *selz*, *tst-1*, *sak*, *chp*, *cna*, and *lukS-PV-lukF-PV*.

**Table 3:** Antimicrobial resistance profile of *S. aureus* and CoNS/*Mammaliicoccus* species isolated from ground meat specimens.

*Staphylococcus* (*Mammaliicoccus*) Species	No. of Isolates	No. of Isolates Showing Resistance to Antimicrobials *^1^ (%)								No. of Isolates Having Resistance Genes *^2^ (%)							
		AMP	GEN	ERY	CLI	CLI-i	LVX	SXT	TET	*blaZ*	*erm*(C)	*lnu*(B)	*aac(6′)-Ie-aph(2″)-Ia*	*ant(4′)-Ia*	*tet*(K)	*tet*(M)	*fosB*
*S. aureus*	10	5 (50) *^3^	0 (0)	2 (20)	0 (0)	2 (20)	1 (10)	0 (0)	0 (0)	5 (50) *^3^	2 (20)	0	0	0	0	0	0
*S. agnetis*	2	1	0	0	0	0	0	0	1	1	0	0	0	0	1	1	0
*S. capitis*	8	1	0	0	0	0	0	1	3	1	0	0	0	0	3	0	5
*S. carnosus*	2	0	0	0	0	0	0	0	0	0	0	0	0	0	0	0	0
*S. chromogenes*	6	2	0	3 *^4^	4 *^4^	0	0	1	4 *^4^	2	3 *^4^	2	0	0	4 *^4^	1	0
*S. cohnii*	5	0	0	0	0	0	0	0	0	0	0	0	0	0	0	0	0
*S. condimenti*	3	0	0	0	0	0	0	0	0	0	0	0	0	0	0	0	0
*S. haemolyticus*	3	0	0	0	0	0	0	0	0	0	0	0	0	0	0	0	0
*S. hyicus*	1	1	0	0	0	0	0	0	1	1	0	0	0	0	1	1	0
*S. kloosii*	1	0	0	0	0	0	0	0	0	0	0	0	0	0	0	0	0
*S. lugdunensis*	1	1	1	0	0	0	0	0	0	1	0	0	1	1	0	0	1
*S. pasteuri*	11	1	0	0	2	0	0	0	2	1	0	0	0	0	0	2	2
*S. rostri*	2	0	0	0	0	0	0	0	2	0	0	0	0	0	0	2	0
*S. saprophyticus*	23	0	0	0	0	0	0	0	3	0	0	0	0	0	2	1	13 *^4^
*S. simulans*	1	0	0	0	0	0	0	0	0	0	0	0	0	0	0	0	0
*S. warneri*	14	13 *^4^	8 *^4^	1	1	1	0	1	3	10 *^4^	1	0	8 *^4^	8 *^4^	3	0	9 *^4^
*S. xylosus*	1	0	0	0	0	0	0	0	0	0	0	0	0	0	0	0	0
*M. lentus*	1	0	0	0	1	0	0	0	1	0	0	0	0	0	1	0	0
*M. sciuri*	20	2	0	0	1	0	0	0	0	2	0	0	0	0	0	0	0
*M. stepanovicii*	3	0	0	0	0	0	0	0	0	0	0	0	0	0	0	0	0
*M. vitulinus*	4	0	0	0	0	0	0	1	1	0	0	0	0	0	1	0	0
Total no. of CoNS/ *Mammaliicoccus*	112	22 (19.6)	9 (8.0)	4 (3.6)	9 (8.0)	1 (0.9)	0 (0)	4 (3.6)	21 (18.8)	19 (17.0)	4 (3.6)	2 (1.8)	9 (8.0)	9 (8.0)	16 (14.3)	8 (7.1)	30 (26.8)

*^1^ Abbreviations: AMP, ampicillin; GEN, gentamicin; ERY, erythromycin; CLI, clindamycin; CLI-i, inducible resistance to clindamycin (confirmed by D-zone test); LVX, levofloxacin; SXT, sulfamethoxazole-trimethoprim; TET, tetracycline. None of the isolates showed resistance to oxacillin, cefoxitin, cefazolin, cefmetazole, flomoxef, imipenem, minocycline, arbekacin, vancomycin, teicoplanin, and linezolid. Presumptive resistance proportion of fosfomycin was described in text. *^2^ None of the isolates had *erm*(A), *erm*(B), *erm*(T), *erm*(Y), *msr*(A), *lnu*(A), *tet*(L), *aph*(3′)-IIIa, *ant*(9)-Ia, *ant*(6)-Ia, *aac*(6′)-Im, *ant*(9)-Ib, *ant*(3″)-Ia, *aph*(2″)-Ic, *aph*(2″)-Id, *optrA*, *fexA* and *cfr*. *^3^ Significantly higher frequency than CoNS (*p* < 0.05). *^4^ Frequency representing significantly higher than other CoNS species (*p* < 0.01).

**Table 4:** *S. capitis* and *S. chromogenes* isolates with and without *tgsB*, *C*, *D*.

Species	Isolate ID	Presence of *tgs* Genes	Other Staphylococcal Species Isolated from the Same Specimen
*S. capitis*	C1	*tgsB*, *C*, *D*	-
	C2	*tgsB*, *C*, *D*	-
	C3	*tgsB*, *C*, *D*	-
	C4	-	-
	C5	-	-
	C46-2	-	*S. warneri*
	C47-2	-	*S. warneri*, *S. lugdunensis*
*S. chromogenes*	M22-2	-	MSSA (ST398), *S. scuiri*
	P12-4	-	MSSA (ST398), *S. pasteuri*, *S. warneri*, *S. haemolyticus*
	C13-1	*tgsC*, *D*	*S. cohnii*, *M. stepanovicii*
	C27-1	*tgsC*, *D*	*S. carnosus*
	C36	-	-
	C52-1	-	-
	M1	-	-

**Table 5:** Co-isolation of *S. aureus* and CoNS/*Mammaliicoccus*.

*S. aureus* Isolate ID	*coa* Type	ST (CC)	CoNS/*Mammaliicoccus* Isolated from the Same Specimen
C26	IIa	ST5 (CC5)	*-*
C35	IIa	ST5 (CC5)	*-*
M3	VIc	ST352 (CC97)	*-*
M4-2	VIIb	ST398	*M. sciuri*
P12-1	VIIb	ST398	*S. chromogenes*, *S. haemolyticus*, *S. pasteuri*, *S. warneri*
M22-1	VIIb	ST398	*S. chromogenes*, *M. sciuri*
C25-1	Xa	ST15	*S. saprophyticus*
M20-1	Xa	ST15	*S. pasteuri*, *S. warneri*, *M. vitulinus*
M2	XIc	ST9 (CC1)	*-*
P7-1	XIc	ST9 (CC1)	*S. rostri*

## Supplementary Materials

The following supporting information can be downloaded at: https://www.mdpi.com/article/10.3390/pathogens11040469/s1, Figure S1: Distribution and frequency of MIC to eight antimicrobials of *S. aureus* and CoNS/Mammaliicoccus isolated from ground meat specimens; Figure S2: Alignment of FosB amino acid sequences analyzed in this study and their identities in clusters of *S. saprophyticus*, *S. warneri*, and *S. capitis*; Table S1: Nucleotide sequence identity (%) of *fosB* among different staphylococcal species/clusters/subclusters; Table S2: Primers to detect 6-TG biosynthesis cluster genes by PCR; Table S3: Primers used to detect *fosB* by PCR for different *Staphylococcus* and *Enterococcus* species; Table S4: Primers used to amplify whole *fosB* ORF to determine its nucleotide sequence; Table S5: Genbank accession numbers of *fosB* gene identified in this study.

## References

[1] Götz F., Bannerman T., Schleifer K.H., Dworkin M., Falkow S., Rosenberg E., Schleifer K.H., Stackebrandt E. (2006). The Genera *Staphylococcus* and *Macrococcus*. The Prokaryotes.

[2] Becker K., Heilmann C., Peters G. (2014). Coagulase-negative staphylococci. Clin. Microbiol. Rev..

[3] Tong S.Y.C., Schaumburg F., Ellington M.J., Corander J., Pichon B., Leendertz F., Bentley S.D., Parkhill J., Holt D.C., Peters G. (2015). Novel staphylococcal species that form part of a *Staphylococcus aureus*-related complex: The non-pigmented *Staphylococcus argenteus* sp. nov. and the non-human primate-associated *Staphylococcus schweitzeri* sp. nov.. Int. J. Syst. Evol. Microbiol..

[4] Chew K.L., Octavia S., Lai D., Lin R.T.P., Teo J.W.P. (2021). *Staphylococcus singaporensis* sp. nov., a new member of the *Staphylococcus aureus* complex, isolated from human clinical specimens. Int. J. Syst. Evol. Microbiol..

[5] Becker K., Schaumburg F., Kearns A., Larsen A.R., Lindsay J.A., Skov R.L., Westh H. (2019). Implications of identifying the recently defined members of the *Staphylococcus aureus* complex *S. argenteus* and *S. schweitzeri*: A position paper of members of the ESCMID Study Group for Staphylococci and Staphylococcal Diseases (ESGS). Clin. Microbiol. Infect..

[6] Heilmann C., Ziebuhr W., Becker K. (2019). Are coagulase-negative staphylococci virulent?. Clin. Microbiol. Infect..

[7] Madhaiyan M., Wirth J.S., Saravanan V.S. (2020). Phylogenomic analyses of the *Staphylococcaceae* family suggest the reclassification of five species within the genus *Staphylococcus* as heterotypic synonyms, the promotion of five subspecies to novel species, the taxonomic reassignment of five *Staphylococcus* species to *Mammaliicoccus* gen. nov., and the formal assignment of *Nosocomiicoccus* to the family *Staphylococcaceae*. Int. J. Syst. Evol. Microbiol..

[8] Argudín M.A., Deplano A., Meghraoui A., Dodémont M., Heinrichs A., Denis O., Nonhoff C., Roisin S. (2017). Bacteria from Animals as a Pool of Antimicrobial Resistance Genes. Antibiotics.

[9] Tsubakishita S., Kuwahara-Arai K., Sasaki T., Hiramatsu K. (2020). Origin and molecular evolution of the determinant of methicillin resistance in staphylococci. Antimicrob. Agents Chemother..

[10] Huber H., Ziegler D., Pflüger V., Vogel G., Zweifel C., Stephan R. (2011). Prevalence and characteristics of methicillin-resistant coagulase-negative staphylococci from livestock, chicken carcasses, bulk tank milk, minced meat, and contact persons. BMC Vet. Res..

[11] Osman K.M., Amer A.M., Badr J.M., Saad A.S. (2015). Prevalence and antimicrobial resistance profile of *Staphylococcus* species in chicken and beef raw meat in Egypt. Foodborne Pathog. Dis..

[12] Can H.Y., Elmalı M., Karagöz A. (2017). Molecular Typing and Antimicrobial Susceptibility of *Staphylococcus aureus* Strains Isolated from Raw Milk, Cheese, Minced Meat, and Chicken Meat Samples. Korean J. Food Sci. Anim. Resour.

[13] Fox A., Pichon B., Wilkinson H., Doumith M., Hill R.L., McLauchlin J., Kearns A.M. (2017). Detection and molecular characterization of Livestock-Associated MRSA in raw meat on retail sale in North West England. Lett. Appl. Microbiol..

[14] Ge B., Mukherjee S., Hsu C.H., Davis J.A., Tran T.T.T., Yang Q., Abbott J.W., Ayers S.L., Young S.R., Crarey E.T. (2017). MRSA and multidrug-resistant *Staphylococcus aureus* in U.S. retail meats, 2010–2011. Food Microbiol..

[15] Tang Y., Larsen J., Kjeldgaard J., Andersen P.S., Skov R., Ingmer H. (2017). Methicillin-resistant and -susceptible *Staphylococcus aureus* from retail meat in Denmark. Int. J. Food Microbiol..

[16] Wu S., Huang J., Wu Q., Zhang J., Zhang F., Yang X., Wu H., Zeng H., Chen M., Ding Y. (2018). *Staphylococcus aureus* Isolated From Retail Meat and Meat Products in China: Incidence, Antibiotic Resistance and Genetic Diversity. Front. Microbiol..

[17] Islam M.A., Parveen S., Rahman M., Huq M., Nabi A., Khan Z.U.M., Ahmed N., Wagenaar J.A. (2019). Occurrence and Characterization of Methicillin Resistant *Staphylococcus aureus* in Processed Raw Foods and Ready-to-Eat Foods in an Urban Setting of a Developing Country. Front. Microbiol..

[18] Kim Y.H., Kim H.S., Kim S., Kim M., Kwak H.S. (2020). Prevalence and Characteristics of Antimicrobial-Resistant *Staphylococcus aureus* and Methicillin-Resistant *Staphylococcus aureus* from Retail Meat in Korea. Food Sci. Anim. Resour.

[19] Tanomsridachchai W., Changkaew K., Changkwanyeun R., Prapasawat W., Intarapuk A., Fukushima Y., Yamasamit N., Flav Kapalamula T., Nakajima C., Suthienkul C. (2021). Antimicrobial Resistance and Molecular Characterization of Methicillin-Resistant *Staphylococcus aureus* Isolated from Slaughtered Pigs and Pork in the Central Region of Thailand. Antibiotics.

[20] Tegegne H.A., Koláčková I., Florianová M., Gelbíčová T., Madec J.Y., Haenni M., Karpíšková R. (2021). Detection and molecular characterisation of methicillin-resistant *Staphylococcus aureus* isolated from raw meat in the retail market. J. Glob. Antimicrob. Resist..

[21] Thwala T., Madoroba E., Basson A., Butaye P. (2021). Prevalence and Characteristics of *Staphylococcus aureus* Associated with Meat and Meat Products in African Countries: A Review. Antibiotics.

[22] Şanlıbaba P. (2022). Prevalence, antibiotic resistance, and enterotoxin production of *Staphylococcus aureus* isolated from retail raw beef, sheep, and lamb meat in Turkey. Int. J. Food Microbiol..

[23] Lee S.I., Kim S.D., Park J.H., Yang S.J. (2020). Species Distribution, Antimicrobial Resistance, and Enterotoxigenicity of Non-aureus Staphylococci in Retail Chicken Meat. Antibiotics.

[24] Kitai S., Shimizu A., Kawano J., Sato E., Nakano C., Kitagawa H., Fujio K., Matsumura K., Yasuda R., Inamoto T. (2005). Prevalence and characterization of *Staphylococcus aureus* and enterotoxigenic *Staphylococcus aureus* in retail raw chicken meat throughout Japan. J. Vet. Med. Sci..

[25] Hiroi M., Kawamori F., Harada T., Sano Y., Miwa N., Sugiyama K., Hara-Kudo Y., Masuda T. (2012). Antibiotic resistance in bacterial pathogens from retail raw meats and food-producing animals in Japan. J. Food Prot..

[26] Sato T., Usui M., Konishi N., Kai A., Matsui H., Hanaki H., Tamura Y. (2017). Closely related methicillin-resistant *Staphylococcus aureus* isolates from retail meat, cows with mastitis, and humans in Japan. PLoS ONE.

[27] Candel F.J., Matesanz David M., Barberán J. (2019). New perspectives for reassessing fosfomycin: Applicability in current clinical practice. Rev. Esp. Quimioter..

[28] Castañeda-García A., Blázquez J., Rodríguez-Rojas A. (2013). Molecular Mechanisms and Clinical Impact of Acquired and Intrinsic Fosfomycin Resistance. Antibiotics.

[29] Fu Z., Liu Y., Chen C., Guo Y., Ma Y., Yang Y., Hu F., Xu X., Wang M. (2016). Characterization of Fosfomycin Resistance Gene, *fosB*, in Methicillin-Resistant *Staphylococcus aureus* Isolates. PLoS ONE.

[30] Lee Y.C., Chen P.Y., Wang J.T., Chang S.C. (2020). Prevalence of fosfomycin resistance and gene mutations in clinical isolates of methicillin-resistant *Staphylococcus aureus*. Antimicrob. Resist. Infect. Control.

[31] Campanile F., Wootton M., Davies L., Aprile A., Mirabile A., Pomponio S., Demetrio F., Bongiorno D., Walsh T.R., Stefani S. (2020). Gold standard susceptibility testing of fosfomycin in *Staphylococcus aureus* and Enterobacterales using a new agar dilution panel^®^. J. Glob. Antimicrob. Resist..

[32] Song Z., Wang X., Zhou X., Jiang S., Li Y., Ahmad O., Qi L., Li P., Li J. (2019). Taxonomic Distribution of FosB in Human-Microbiota and Activity Comparison of Fosfomycin Resistance. Front. Microbiol..

[33] Chin D., Goncheva M.I., Flannagan R.S., Deecker S.R., Guariglia-Oropeza V., Ensminger A.W., Heinrichs D.E. (2021). Coagulase-negative staphylococci release a purine analog that inhibits *Staphylococcus aureus* virulence. Nat. Commun..

[34] Torki Baghbaderani Z., Shakerian A., Rahimi E. (2020). Phenotypic and Genotypic Assessment of Antibiotic Resistance of *Staphylococcus aureus* Bacteria Isolated from Retail Meat. Infect. Drug Resist..

[35] Buyukcangaz E., Velasco V., Sherwood J.S., Stepan R.M., Koslofsky R.J., Logue C.M. (2013). Molecular typing of *Staphylococcus aureus* and methicillin-resistant *S. aureus* (MRSA) isolated from animals and retail meat in North Dakota, United States. Foodborne Pathog. Dis..

[36] Jackson C.R., Davis J.A., Barrett J.B. (2013). Prevalence and characterization of methicillin-resistant *Staphylococcus aureus* isolates from retail meat and humans in Georgia. J. Clin. Microbiol..

[37] Thapaliya D., Forshey B.M., Kadariya J., Quick M.K., Farina S., O’ Brien A., Nair R., Nworie A., Hanson B., Kates A. (2017). Prevalence and molecular characterization of *Staphylococcus aureus* in commercially available meat over a one-year period in Iowa, USA. Food Microbiol..

[38] Anjum M.F., Marco-Jimenez F., Duncan D., Marín C., Smith R.P., Evans S.J. (2019). Livestock-Associated Methicillin-Resistant *Staphylococcus aureus* From Animals and Animal Products in the UK. Front. Microbiol..

[39] Li H., Tang T., Stegger M., Dalsgaard A., Liu T., Leisner J.J. (2021). Characterization of antimicrobial-resistant *Staphylococcus aureus* from retail foods in Beijing, China. Food Microbiol..

[40] Verkade E., Kluytmans J. (2014). Livestock-associated *Staphylococcus aureus* CC398: Animal reservoirs and human infections. Infect. Genet. Evol..

[41] Hata E., Katsuda K., Kobayashi H., Uchida I., Tanaka K., Eguchi M. (2010). Genetic variation among *Staphylococcus aureus* strains from bovine milk and their relevance to methicillin-resistant isolates from humans. J. Clin. Microbiol..

[42] Dastmalchi Saei H., Panahi M. (2020). Genotyping and antimicrobial resistance of *Staphylococcus aureus* isolates from dairy ruminants: Differences in the distribution of clonal types between cattle and small ruminants. Arch. Microbiol..

[43] Achek R., El-Adawy H., Hotzel H., Hendam A., Tomaso H., Ehricht R., Neubauer H., Nabi I., Hamdi T.M., Monecke S. (2021). Molecular Characterization of *Staphylococcus aureus* Isolated from Human and Food Samples in Northern Algeria. Pathogens.

[44] Rainard P., Corrales J.C., Barrio M.B., Cochard T., Poutrel B. (2003). Leucotoxic activities of *Staphylococcus aureus* strains isolated from cows, ewes, and goats with mastitis: Importance of LukM/LukF’-PV leucotoxin. Clin. Diagn. Lab. Immunol..

[45] Chajęcka-Wierzchowska W., Zadernowska A., Nalepa B., Sierpińska M., Łaniewska-Trokenheim L. (2015). Coagulase-negative staphylococci (CoNS) isolated from ready-to-eat food of animal origin—Phenotypic and genotypic antibiotic resistance. Food Microbiol..

[46] Guran H.S., Kahya S. (2015). Species Diversity and Pheno- and Genotypic Antibiotic Resistance Patterns of Staphylococci Isolated from Retail Ground Meats. J. Food Sci..

[47] Jans C., Merz A., Johler S., Younan M., Tanner S.A., Kaindi D.W.M., Wangoh J., Bonfoh B., Meile L., Tasara T. (2017). East and West African milk products are reservoirs for human and livestock-associated *Staphylococcus aureus*. Food Microbiol..

[48] Xu W., Chen T., Wang H., Zeng W., Wu Q., Yu K., Xu Y., Zhang X., Zhou T. (2020). Molecular Mechanisms and Epidemiology of Fosfomycin Resistance in *Staphylococcus aureus* Isolated From Patients at a Teaching Hospital in China. Front. Microbiol..

[49] DiCicco M., Weese S., Neethirajan S., Rousseau J., Singh A. (2014). Fosfomycin susceptibility of canine methicillin-resistant *Staphylococcus pseudintermedius* isolates. Res. Vet. Sci..

[50] Argudín M.A., Vanderhaeghen W., Butaye P. (2015). Diversity of antimicrobial resistance and virulence genes in methicillin-resistant non-*Staphylococcus aureus* staphylococci from veal calves. Res. Vet. Sci..

[51] Higashide M., Kuroda M., Omura C.T., Kumano M., Ohkawa S., Ichimura S., Ohta T. (2008). Methicillin-resistant *Staphylococcus saprophyticus* isolates carrying staphylococcal cassette chromosome *mec* have emerged in urogenital tract infections. Antimicrob. Agents Chemother..

[52] Nakaminami H., Noguchi N., Nishijima S., Kurokawa I., Sasatsu M. (2008). Characterization of the pTZ2162 encoding multidrug efflux gene qacB from *Staphylococcus aureus*. Plasmid.

[53] He T., Wang Y., Schwarz S., Zhao Q., Shen J., Wu C. (2014). Genetic environment of the multi-resistance gene *cfr* in methicillin-resistant coagulase-negative staphylococci from chickens, ducks, and pigs in China. Int. J. Med. Microbiol..

[54] Liu B.H., Lei C.W., Zhang A.Y., Pan Y., Kong L.H., Xiang R., Wang Y.X., Yang Y.X., Wang H.N. (2017). Colocation of the Multiresistance Gene *cfr* and the Fosfomycin Resistance Gene *fosD* on a Novel Plasmid in *Staphylococcus arlettae* from a Chicken Farm. Antimicrob. Agents Chemother..

[55] De Freire Bastos M.D.C., Miceli de Farias F., Carlin Fagundes P., Varella Coelho M.L. (2020). Staphylococcins: An update on antimicrobial peptides produced by staphylococci and their diverse potential applications. Appl. Microbiol. Biotechnol..

[56] Hirose M., Aung M.S., Fukuda A., Yahata S., Fujita Y., Saitoh M., Hirose Y., Urushibara N., Kobayashi N. (2021). Antimicrobial Resistance and Molecular Epidemiological Characteristics of Methicillin-Resistant and Susceptible Staphylococcal Isolates from Oral Cavity of Dental Patients and Staff in Northern Japan. Antibiotics.

[57] Zhang K., McClure J.A., Elsayed S., Louie T., Conly J.M. (2008). Novel multiplex PCR assay for simultaneous identification of community-associated methicillin-resistant *Staphylococcus aureus* strains USA300 and USA400 and detection of mecA and Panton-Valentine leukocidin genes, with discrimination of *Staphylococcus aureus* from coagulase-negative staphylococci. J. Clin. Microbiol..

[58] Zhang D.F., Xu X., Song Q., Bai Y., Zhang Y., Song M., Shi C., Shi X. (2016). Identification of *Staphylococcus argenteus* in Eastern China based on a nonribosomal peptide synthetase (NRPS) gene. Future Microbiol..

[59] Clinical and Laboratory Standards Institute (CLSI) (2018). Performance Standards for Antimicrobial Susceptibility Testing.

[60] The European Committee on Antimicrobial Susceptibility Testing (EUCAST) (2021). Breakpoint Tables for Interpretation of MICs and Zone Diameters. Version 11.0. http://www.eucast.org.

[61] Watanabe A., Yanagihara K., Matsumoto T., Kohno S., Aoki N., Oguri T., Sato J., Muratani T., Yagisawa M., Ogasawara K. (2012). National surveillance of bacterial respiratory pathogens conducted by the Surveillance Committee of Japanese Society of Chemotherapy, Japanese Association for Infectious Diseases. J. Infect. Chemother..

[62] Aung M.S., Urushibara N., Kawaguchiya M., Sumi A., Shinagawa M., Takahashi S., Kobayashi N. (2019). Clonal Diversity and Genetic Characteristics of Methicillin-Resistant *Staphylococcus aureus* Isolates from a Tertiary Care Hospital in Japan. Microb. Drug Resist..

[63] Roy S., Aung M.S., Paul S.K., Ahmed S., Haque N., Khan E.R., Barman T.K., Islam A., Abedin S., Sultana C. (2020). Drug Resistance Determinants in Clinical Isolates of *Enterococcus faecalis* in Bangladesh: Identification of Oxazolidinone Resistance Gene optrA in ST59 and ST902 Lineages. Microorganisms.

[64] Enright M.C., Day N.P., Davies C.E., Peacock S.J., Spratt B.G. (2000). Multilocus sequence typing for characterization of methicillin-resistant and methicillin-susceptible clones of *Staphylococcus aureus*. J. Clin. Microbiol..

[65] Shopsin B., Gomez M., Montgomery S.O., Smith D.H., Waddington M., Dodge D.E., Bost D.A., Riehman M., Naidich S., Kreiswirth B.N. (1999). Evaluation of protein A gene polymorphic region DNA sequencing for typing of *Staphylococcus aureus* strains. J. Clin. Microbiol..

[66] Aung M.S., Urushibara N., Kawaguchiya M., Ito M., Habadera S., Kobayashi N. (2020). Prevalence and Genetic Diversity of Staphylococcal Enterotoxin (-Like) Genes *sey*, *selw*, *selx*, *selz*, *sel26* and *sel27* in Community-Acquired Methicillin-Resistant *Staphylococcus aureus*. Toxins.

