# Supplementary material for: Prevalence and Antimicrobial Resistance of Staphylococcus aureus and Coagulase-Negative Staphylococcus/Mammaliicoccus from Retail Ground Meat: Identification of Broad Genetic Diversity in Fosfomycin Resistance Gene fosB
Source: Pathogens. 2022 Apr 14;11(4):469. doi: 10.3390/pathogens11040469 (PMC9031665; doi:10.3390/pathogens11040469)
Supplement: Supplementary file 1 [file pathogens-11-00469-s001.zip › Supplementary Figure S2.pdf]

(a)

```
S.saprophyticus-C57-1      MIQSINHVTYSVSDISKSINFYKDILKAKILVESDKTAYFTLGGLWLALNEEKDTPRNEI   60
S.saprophyticus-C561       MIQSINHVTYSVSDISKSINFYKDILKAKILVESDKTAYFTLGGLWLALNEEKDTPRNEI   60
S.saprophyticus-C58-1      MIQSINHVTYSVSDISKSINFYKDILKAKILVESDKTAYFTLGGLWLALNEEKDTPRNEI   60
S.saprophyticus-C55-2      MIQSINHVTYSVSDIKASITFYKDILKANILVESDKTAYFTVGGLWLALNEEKDIPRNEI   60
S.saprophyticus-M25-2      MIQSINHVTYSVSDIKASITFYKDILKANILVESDKTAYFTVGGLWLALNEEKDIPRNEI   60
S.saprophyticus-C44-2      MIQSINHVTYSVSDIKASITFYKDILKANILVESDKTAYFTVGGLWLALNEEKDIPRNEI   60
S.saprophyticus-C60-1      MIQTINHVTYSVSDIKASITFYKDILKANILVESDKTAYFTVGGLWLALNEEKDIPRNEI   60
S.saprophyticus-C39-2      MIQTINHVTYSVSDIKASITFYKDILKANILVESDKTAYFTVGGLWLALNEEKDIPRNEI   60
S.saprophyticus-M11-1      MIQTINHVTYSVSDIKASITFYKDILKANILVESDKTAYFTVGGLWLALNEEKDIPRNEI   60
S.saprophyticus-P6-1       MIQTINHVTYSVSDIKASITFYKDILKANILVESDKTAYFTVGGLWLALNEEKDIPRNEI   60
S.saprophyticus-P18-2      LIQSINHVTYSVSDINNSIAFYKDVLKAKVLVESDKTAYFTIGGLWLALNEEKDISRNEI   60
S.saprophyticus-M17-3      MIQSINHVTYSVSDIKTSIAFYQDILKAEILVESDKTVYFTLGGLWLALNEEKDIPRNEI   60
S.saprophyticus-UTI-035    MIQSINHVTYSVSDIKASITFYKDILKANILVESDKTAYFTVGGLWLALNEEKDIPRNEI   60
                           :**:***********. ** **:*:***::*******.***:************  ****

S.saprophyticus-C57-1      QYSYTHMAFKIDESEFDEWYQWLKDNYVNILEGRSRDLRDKKSIYFTDPDGHKLELHTGT   120
S.saprophyticus-C561       QYSYTHMAFKIDESEFDEWYQWLKDNYVNILEGRSRDLRDKKSIYFTDPDGHKLELHTGT   120
S.saprophyticus-C58-1      QYSYTHMAFKIDESEFDEWYQWLKDNYVNILEGRSRDLRDKKSIYFTDPDGHKLELHTGT   120
S.saprophyticus-C55-2      AYSYTHMAFTIDESEFDEWYQWYKDNNVNILEGRNRDVRDKNSIYFTDPDVHKLELHTGT   120
S.saprophyticus-M25-2      AYSYTHMAFTIDESEFDEWYQWYKDNNVNILEGRNRDVRDKNSIYFTDPDVHKLELHTGT   120
S.saprophyticus-C44-2      AYSYTHMAFTIDESEFDEWYQWYKDNNVNILEGRNRDVRDKNSIYFTDPDVHKLELRTGT   120
S.saprophyticus-C60-1      AYSYTHMAFTIDESEFDEWYQWYKDNNVNILEGRNRDVRDKNSIYFTDPDVHKLELHTGT   120
S.saprophyticus-C39-2      AYSYTHMAFTIDESEFDEWYQWYKDNNVNILEGRNRDVRDKNSIYFTDPDVHKLELHTGT   120
S.saprophyticus-M11-1      AYSYTHMAFTIDESEFDEWYQWYKDNNVNILEGRNRDVRDKNSIYFTDPDVHKLELHTGT   120
S.saprophyticus-P6-1       AYSYTHMAFTIDESEFDEWYQWYKDNNVNILEGRNRDVRDKNSIYFTDPDVHKLELRTGT   120
S.saprophyticus-P18-2      QYSYTHMAFTINENEFDEWYQWLKDNNVNILEGRNRDIRDKQSIYFTDPDGHKFELHTGT   120
S.saprophyticus-M17-3      QYSYTHMAFTIDENEFEEWYQWLKDNDVNILEGRNRDIREKKSIYFTDPDGHKLELHTGT   120
S.saprophyticus-UTI-035    AYSYTHMAFTIDESEFDEWYQWYKDNNVNILEGRNRDVRDKNSIYFTDPDVHKLELHTGT   120
                            ********.*:*.**:***** *** *******.**:*:*:******** **:**:***

S.saprophyticus-C57-1      LQDRLDYYKEEKSHMKFYI   139
S.saprophyticus-C561       LQDRLDYYKEEKSHMKFYI   139
S.saprophyticus-C58-1      LQDRLDYYKEEKSHMKFYI   139
S.saprophyticus-C55-2      LQDRLDYYKEEKPHMKFYI   139
S.saprophyticus-M25-2      LQDRLDYYKEEKPHMKFYK   139
S.saprophyticus-C44-2      LQDRLDYYKEEKPHMKFYK   139
S.saprophyticus-C60-1      LQDRLDYYKEEKPHMKFYI   139
S.saprophyticus-C39-2      LQDRLDYYKEEKPHMKFYK   139
S.saprophyticus-M11-1      LQDRLDYYKEEKPHMKFYK   139
S.saprophyticus-P6-1       LQDRLDYYKEEKPHMKFYK   139
S.saprophyticus-P18-2      LQDRLNYYKEEKPHMKFYI   139
S.saprophyticus-M17-3      LQDRLNYYKEEKPHMKFYI   139
S.saprophyticus-UTI-035    LQDRLDYYKEEKPHMNFYK   139
                           *****:****** **:**
```

No.	1	2	3	4	5	6	7	8	9	10	11	12	13
1: S.saprophyticus-C57-1	100.00	100.00	100.00	89.21	87.77	88.49	87.77	88.49	87.77	87.77	87.05	86.33	88.49
2: S.saprophyticus-C561	100.00	100.00	100.00	89.21	87.77	88.49	87.77	88.49	87.77	87.77	87.05	86.33	88.49
3: S.saprophyticus-C58-1	100.00	100.00	100.00	89.21	87.77	88.49	87.77	88.49	87.77	87.77	87.05	86.33	88.49
4: S.saprophyticus-C55-2	89.21	89.21	89.21	100.00	98.56	99.28	98.56	99.28	98.56	98.56	97.84	87.05	88.49
5: S.saprophyticus-UTI-035	87.77	87.77	87.77	98.56	100.00	99.28	98.56	97.84	98.56	98.56	97.84	85.61	87.05
6: S.saprophyticus-M25-2	88.49	88.49	88.49	99.28	99.28	100.00	99.28	98.56	99.28	99.28	98.56	86.33	87.77
7: S.saprophyticus-C44-2	87.77	87.77	87.77	98.56	98.56	99.28	100.00	97.84	98.56	98.56	99.28	85.61	87.05
8: S.saprophyticus-C60-1	88.49	88.49	88.49	99.28	97.84	98.56	97.84	100.00	99.28	99.28	98.56	86.33	87.77
9: S.saprophyticus-C39-2	87.77	87.77	87.77	98.56	98.56	99.28	98.56	99.28	100.00	100.00	99.28	85.61	87.05
10: S.saprophyticus-M11-1	87.77	87.77	87.77	98.56	98.56	99.28	98.56	99.28	100.00	100.00	99.28	85.61	87.05
11: S.saprophyticus-P6-1	87.05	87.05	87.05	97.84	97.84	98.56	99.28	98.56	99.28	99.28	100.00	84.89	86.33
12: S.saprophyticus-P18-2	86.33	86.33	86.33	87.05	85.61	86.33	85.61	86.33	85.61	85.61	84.89	100.00	88.49
13: S.saprophyticus-M17-3	88.49	88.49	88.49	88.49	87.05	87.77	87.05	87.77	87.05	87.05	86.33	88.49	100.00

(b)

```
S.warneri-C33-1          MIQAVNHVTYSVSDIKKSIEFYKNILKAKILLESDKTAYFTIGGLWLALNEESEIPRNEI    60
S.lugdunensis-C47-3      MIQSVNHVTYSVSDIKKSVEFYKNILKAKILLESDKTAYFTIGGLWLGLNEESEIPRNEI    60
S.pasteiri-C34-2         MIQAVNHVTYSVSDIKKSVEFYKNILKAKILLESDKTAYFTIGGLWLGLNEESEIPRNEI    60
S.pasteiri-C65-1         MIQAVNHVTYSVSDIKKSVEFYKNILKAKILLESDKTAYFTIGGLWLGLNEESEIPRNEI    60
S.warneri-C6             MIQAVNHVTYSVSDIKKSVEFYKNILKAKILLESDKTAYFTIGGLWLGLNEESEIPRNEI    60
S.warneri-C46-1          MIQAVNHVTYSVSDIKKSVEFYKNILKAKILLESDKTAYFTIGGLWLGLNEESEIPRNEI    60
S.warneri-C47-1          MIQAVNHVTYSVSDIKKSVEFYKNILKAKILLESDKTAYFTIGGLWLGLNEESEIPRNEI    60
S.warneri-C49-1          MIQAVNHVTYSVSDIKKSVEFYKNILKAKILLESDKTAYFTIGGLWLGLNEESEIPRNEI    60
S.warneri-C50-2          MIQAVNHVTYSVSDIKKSVEFYKNILKAKILLESDKTAYFTIGGLWLGLNEESEIPRNEI    60
S.warneri-C51-2          MIQAVNHVTYSVSDIKKSVEFYKNILKAKILLESDKTAYFTIGGLWLGLNEESEIPRNEI    60
S.warneri-C63            MIQAVNHVTYSVSDIKKSVEFYKNILKAKILLESDKTAYFTIGGLWLGLNEESEIPRNEI    60
S.warneri-M21-3          MIQAVNHVTYSVSDIKKSVEFYKNILKAKILLESDKTAYFTIGGLWLGLNEESEIPRNEI    60
S.warneri-DE0454         MIQAVNHVTYSVSDIKKSVEFYKNILKAKILLESDKTAYFTIGGLWLALNEESEIPRNEI    60
                         ***:**************:*****************************.***********

S.warneri-C33-1          HYSYTHMAFSIKENDFDEWYDWLKQNHVNILEGRPRDVRDKKSIYFTDPDGHKLELHTGS    120
S.lugdunensis-C47-3      HYSYTHMAFSIKENDFDEWYDWLKQNHVNILEGRPRDVRDKKSIYFTDPDGHKLELHTGS    120
S.pasteiri-C34-2         HYSYTHMAFSIKENDFDEWYDWLKQNHVNILEGRPRDVRDKKSIYFTDPDGHKLELHTGS    120
S.pasteiri-C65-1         HYSYTHMAFSIKENDFDEWYDWLKQNHVNILEGRPRDVRDKKSIYFTDPDGHKLELHTGS    120
S.warneri-C6             HYSYTHMAFSIKENDFDEWYDWLKQNHVNILEGRPRDVRDKKSIYFTDPDGHKLELHTGS    120
S.warneri-C46-1          HYSYTHMAFSIKENDFDEWYDWLKQNHVNILEGRPRDVRDKKSIYFTDPDGHKLELHTGS    120
S.warneri-C47-1          HYSYTHMAFSIKENDFDEWYDWLKQNHVNILEGRPRDVRDKKSIYFTDPDGHKLELHTGS    120
S.warneri-C49-1          HYSYTHMAFSIKENDFDEWYDWLKQNHVNILEGRPRDVRDKKSIYFTDPDGHKLELHTGS    120
S.warneri-C50-2          HYSYTHMAFSIKENDFDEWYDWLKQNHVNILEGRPRDVRDKKSIYFTDPDGHKLELHTGS    120
S.warneri-C51-2          HYSYTHMAFSIKENDFDEWYDWLKQNHVNILEGRPRDVRDKKSIYFTDPDGHKLELHTGS    120
S.warneri-C63            HYSYTHMAFSIKENDFDEWYDWLKQNHVNILEGRPRDVRDKKSIYFTDPDGHKLELHTGS    120
S.warneri-M21-3          HYSYTHMAFSIKENDFDEWYDWLKQNHVNILEGRPRDVRDKKSIYFTDPDGHKLELHTGS    120
S.warneri-DE0454         HYSYTHMAFSIKENDFDEWYDWLKQNHVNILEGRPRDVRDKKSIYFTDPDGHKLELHTGC    120
                         ***********************************************************.

S.warneri-C33-1          LQDRMNYYKDEKSHIKFYE    139
S.lugdunensis-C47-3      LQDRMNYYKDEKPHIKFNE    139
S.pasteiri-C34-2         LQDRMNYYKDEKPHIKFYE    139
S.pasteiri-C65-1         LQDRMNYYKDEKPHIKFYE    139
S.warneri-C6             LQDRMNYYKDEKPHIKFYE    139
S.warneri-C46-1          LQDRMNYYKDEKPHIKFYE    139
S.warneri-C47-1          LQDRMNYYKDEKPHIKFYE    139
S.warneri-C49-1          LQDRMNYYKDEKPHIKFYE    139
S.warneri-C50-2          LQDRMNYYKDEKPHIKFYE    139
S.warneri-C51-2          LQDRMNYYKDEKPHIKFYE    139
S.warneri-C63            LQDRMNYYKDEKPHIKFYE    139
S.warneri-M21-3          LQDRMNYYKDEKPHIKFYE    139
S.warneri-DE0454         LQDRMNYYKDEKPHIKFYE    139
                         ************ **** *
```

No.	1	2	3	4	5	6	7	8	9	10	11	12	13
1: S.warneri-C33-1	100.00	96.40	97.84	97.84	97.84	97.84	97.84	97.84	97.84	97.84	97.84	97.84	97.84
2: S.lugdunensis-C47-3	96.40	100.00	97.12	98.56	98.56	98.56	98.56	98.56	98.56	98.56	98.56	98.56	98.56
3: S.warneri-DE0454	97.84	97.12	100.00	98.56	98.56	98.56	98.56	98.56	98.56	98.56	98.56	98.56	98.56
4: S.pasteiri-C34-2	97.84	98.56	98.56	100.00	100.00	100.00	100.00	100.00	100.00	100.00	100.00	100.00	100.00
5: S.pasteiri-C65-1	97.84	98.56	98.56	100.00	100.00	100.00	100.00	100.00	100.00	100.00	100.00	100.00	100.00
6: S.warneri-C6	97.84	98.56	98.56	100.00	100.00	100.00	100.00	100.00	100.00	100.00	100.00	100.00	100.00
7: S.warneri-C46-1	97.84	98.56	98.56	100.00	100.00	100.00	100.00	100.00	100.00	100.00	100.00	100.00	100.00
8: S.warneri-C47-1	97.84	98.56	98.56	100.00	100.00	100.00	100.00	100.00	100.00	100.00	100.00	100.00	100.00
9: S.warneri-C49-1	97.84	98.56	98.56	100.00	100.00	100.00	100.00	100.00	100.00	100.00	100.00	100.00	100.00
10: S.warneri-C50-2	97.84	98.56	98.56	100.00	100.00	100.00	100.00	100.00	100.00	100.00	100.00	100.00	100.00
11: S.warneri-C51-2	97.84	98.56	98.56	100.00	100.00	100.00	100.00	100.00	100.00	100.00	100.00	100.00	100.00
12: S.warneri-C63	97.84	98.56	98.56	100.00	100.00	100.00	100.00	100.00	100.00	100.00	100.00	100.00	100.00
13: S.warneri-M21-3	97.84	98.56	98.56	100.00	100.00	100.00	100.00	100.00	100.00	100.00	100.00	100.00	100.00

(c)

```
S.capitis-C1        MNIQGINHICFSVSNLVESIKFYRDVLKGELLVSGKTTAYFDIGGLWVALNEEVNVPRTE   60
S.capitis-C46-2     MNIQGINHICFSVSNLVESIKFYRDVLKGELLVSGKTTAYFDIGGLWVALNEEVNVPRTE   60
S.capitis-C47-2     MNIQGINHICFSVSNLVESIKFYRDVLKGELLVSGKTTAYFDIGGLWVALNEEVNVPRTE   60
S.capitis-C55-4     MNIQGINHICFSVSNLVESIKFYRDVLKGELLVSGKTTAYFDIGGLWVALNEEVNVPRTE   60
S.capitis-C2        MDIQGFNHICFSVSNLVESIKFYRDVLKGELLVSGKTTAYFDIGGLWVALNEEVNVPRTE   60
S.capitis-BN2       MNIQGINHICFSVSNLVESIKFYRDVLKGELLVSGKTTAYFDIGGLWVALNEEVNVPRTE   60
                    *:***:******************************************************

S.capitis-C1        IKYSYTHIAFSINENEFEEWYKWLQKHNVNILEGRKRSHQDKKSIYFTDPDGHKLELHTG   120
S.capitis-C46-2     IKYSYTHIAFSINENEFEEWYKWLQKHNVNILEGRKRSHQDKKSIYFTDPDGHKLELHTG   120
S.capitis-C47-2     IKYSYTHIAFSINENEFEEWYKWLQKHNVNILEGRKRSHQDKKSIYFTDPDGHKLELHTG   120
S.capitis-C55-4     IKYSYTHIAFSINENEFEEWYKWLQKHNVNILEGRKRSHQDKKSIYFTDPDGHKLELHTG   120
S.capitis-C2        IKYSYTHIAFSINENEFEEWYKWLQKYNVNILEGRKRSHQDKKSIYFTDPDGHKLELHTG   120
S.capitis-BN2       IKYSYTHIAFSINENEFEEWYKWLQKHNVNILEGRKRSHQDKKSIYFTDPDGHKLELHTG   120
                    **************************:*********************************

S.capitis-C1        TLNDRLDYYKKEKTHMTFYY 140
S.capitis-C46-2     TLNDRLDYYKKEKTHMTFYY 140
S.capitis-C47-2     TLNDRLDYYKKEKTHMTFYY 140
S.capitis-C55-4     TLNDRLDYYKKEKTHMTFYY 140
S.capitis-C2        TLNDRLDYYKKEKTHMTFYY 140
S.capitis-BN2       TLNDRLDYYKKEKTHMTFYY 140
                    ********************
```

No.	1	2	3	4	5	6
1: S.capitis-BN2	100.00	100.00	100.00	100.00	100.00	97.86
2: S.capitis-C1	100.00	100.00	100.00	100.00	100.00	97.86
3: S.capitis-C46-2	100.00	100.00	100.00	100.00	100.00	97.86
4: S.capitis-C47-2	100.00	100.00	100.00	100.00	100.00	97.86
5: S.capitis-C55-4	100.00	100.00	100.00	100.00	100.00	97.86
6: S.capitis-C2	97.86	97.86	97.86	97.86	97.86	100.00

Supplementary Figure S2 Alignment of FosB amino acid sequences analyzed in this study and their identities in clusters of *S. saprophyticus* (a), *S. warneri* (b), and *S. capitis* (c). Reference sequences were added in each analysis: *S. saprophyticus* strain UTI-035, *S. warneri* strain DE0454, and *S. capitis* strain BN2 (GenBank accession nos. CP054434, VDRS01000003, and CP042341, respectively). Asterisk (*) indicates identical amino acid, while colon (:) and period (.) indicate conservation between amino acid groups of strongly and weakly similar properties, respectively. Sequence identity among isolates/strains is shown below the alignment.
