# Supplementary material for: Prevalence and Antimicrobial Resistance of Staphylococcus aureus and Coagulase-Negative Staphylococcus/Mammaliicoccus from Retail Ground Meat: Identification of Broad Genetic Diversity in Fosfomycin Resistance Gene fosB
Source: Pathogens. 2022 Apr 14;11(4):469. doi: 10.3390/pathogens11040469 (PMC9031665; doi:10.3390/pathogens11040469)
Supplement: Supplementary file 1 [file pathogens-11-00469-s001.zip › Supplementary Table S1.pdf]

Table S1 Nucleotide sequence identity (%) of *fosB* among different staphylococcal species/clusters/subclusters

Cluster	*S. aureus* I				*S. warneri*				*S. saprophyticus*															*S. aureus* II						*S. epidermidis*		*S. lugdunensis*			*S. capitis*						
Subcluster (SC)					1	2			3		2		1											1			2								2		1				
Strain / isolate ID																																									
1: S.aureus-SA1278_fosB6_	100.00	99.29	99.29	99.05	73.33	73.10	73.57	74.05	73.33	73.33	74.05	73.57	74.05	73.81	73.57	74.05	73.81	75.24	74.29	73.81	73.10	76.19	78.33	69.29	69.52	69.52	70.71	70.48	64.05	67.62	67.38	70.71	70.95	70.71	72.55	71.60	71.90	72.08	72.32	72.32	72.32
2: S.aureus-SA0406_fosB4_	99.29	100.00	99.52	99.29	73.10	72.86	73.33	73.81	73.57	73.57	74.29	73.81	74.29	74.05	73.81	74.29	74.05	75.24	74.52	74.05	73.33	76.19	78.33	69.05	69.29	69.29	70.71	70.48	64.29	67.14	66.90	70.71	70.95	70.71	72.79	71.84	71.67	71.84	72.08	72.08	72.08
3: S.aureus-au-HS166_fosB1_	99.29	99.52	100.00	99.76	73.57	73.33	73.81	74.29	73.57	73.57	74.29	73.81	74.29	74.05	73.81	74.29	74.05	75.48	74.52	74.05	73.33	76.43	78.57	69.52	69.76	69.76	70.95	70.71	64.29	67.38	67.14	71.19	71.43	71.19	73.03	72.08	72.14	72.32	72.55	72.55	72.55
4: S.aureus-SA1280_fosB5_	99.05	99.29	99.76	100.00	73.81	73.57	74.05	74.52	73.81	73.81	74.52	74.05	74.52	74.29	74.05	74.52	74.29	75.71	74.76	74.29	73.57	76.67	78.81	69.52	69.76	69.76	70.71	70.48	64.52	67.62	67.38	71.43	71.67	71.43	73.27	72.32	72.38	72.55	72.79	72.79	72.79
5: S.pasteuri-DSM10656	73.33	73.10	73.57	73.81	100.00	92.38	91.90	91.90	79.05	79.05	80.48	78.33	79.76	79.76	79.76	79.76	79.52	80.48	79.76	78.81	80.24	80.95	81.19	67.14	67.14	66.90	70.24	70.48	66.19	67.86	67.62	70.00	70.24	70.00	67.54	67.54	69.29	69.21	69.45	69.45	69.69
6: S.warneri-C33-1	73.10	72.86	73.33	73.57	92.38	100.00	97.14	97.62	79.52	79.52	79.76	78.10	79.29	79.52	79.29	79.29	79.05	79.76	80.24	77.62	78.81	79.76	80.48	66.43	66.43	66.19	69.52	70.24	66.67	67.14	66.90	71.19	70.95	71.19	68.26	68.26	69.05	68.97	69.21	69.21	69.45
7: S.lugdunensis-C47-3	73.57	73.33	73.81	74.05	91.90	97.14	100.00	99.52	79.76	79.76	79.29	77.38	79.05	79.05	79.05	79.05	78.81	79.52	79.76	77.14	78.10	79.29	79.52	65.95	65.95	65.71	69.52	70.24	65.71	66.67	66.43	70.00	70.24	70.00	67.54	67.54	68.57	68.50	68.74	68.74	68.97
8: S.warneri-C63	74.05	73.81	74.29	74.52	91.90	97.62	99.52	100.00	79.76	79.76	79.29	77.38	79.05	79.29	79.05	79.05	78.81	79.52	79.76	77.14	78.10	79.29	79.52	65.95	65.95	65.71	69.52	70.24	66.19	66.90	66.67	70.48	70.71	70.48	68.02	68.02	69.05	68.97	69.21	69.21	69.45
9: S.saprophyticus-ATCC15305	73.33	73.57	73.57	73.81	79.05	79.52	79.76	79.76	100.00	100.00	89.05	86.90	86.67	86.67	86.67	86.67	86.43	88.33	87.62	85.95	85.71	86.67	86.90	71.90	71.67	71.43	74.05	74.29	65.48	66.67	66.43	67.62	67.86	67.62	65.67	65.21	66.30	68.74	69.45	69.45	69.21
10: S.saprophyticus-FDAARGOS_355	73.33	73.57	73.57	73.81	79.05	79.52	79.76	79.76	100.00	100.00	89.05	86.90	86.67	86.67	86.67	86.67	86.43	88.33	87.62	85.95	85.71	86.67	86.90	71.90	71.67	71.43	74.05	74.29	65.48	66.67	66.43	67.62	67.86	67.62	65.67	65.21	66.67	68.74	69.45	69.45	69.21
11: S.saprophyticus-C57-1	74.05	74.29	74.29	74.52	80.48	79.76	79.29	79.29	89.05	89.05	100.00	87.86	89.76	89.29	89.29	89.29	89.05	89.52	88.33	88.10	89.76	90.00	88.57	70.95	70.95	70.71	74.29	74.76	65.00	67.14	66.90	69.29	69.52	69.29	67.78	67.78	70.71	70.88	71.36	71.36	71.12
12: S.equorum-KS1039	73.57	73.81	73.81	74.05	78.33	78.10	77.38	77.38	86.90	86.90	87.86	100.00	89.76	89.29	89.29	89.29	89.05	88.10	90.48	89.05	90.24	90.71	89.05	70.24	70.24	70.24	74.29	74.76	66.90	66.43	66.19	69.76	69.52	69.76	67.06	66.59	69.52	69.69	70.17	70.17	69.93
13: S.saprophyticus-C55-2	74.05	74.29	74.29	74.52	79.76	79.29	79.05	79.05	86.67	86.67	89.76	89.76	100.00	99.52	99.52	99.52	99.29	90.48	88.57	89.76	88.33	89.29	90.71	71.90	71.90	71.90	75.00	75.48	66.43	67.86	67.62	69.52	69.29	69.52	69.21	68.74	70.71	70.88	71.36	71.36	71.12
14: S.saprophyticus-M11-1	73.81	74.05	74.05	74.29	79.76	79.52	79.05	79.29	86.67	86.67	89.29	89.29	99.52	100.00	99.52	99.52	99.29	90.00	88.10	89.29	87.86	88.81	90.24	71.90	71.90	71.90	75.00	75.48	66.19	68.33	68.10	69.29	69.05	69.29	69.45	68.97	70.95	71.12	71.60	71.60	71.36
15: S.saprophyticus-C44-2	73.57	73.81	73.81	74.05	79.76	79.29	79.05	79.05	86.67	86.67	89.29	89.29	99.52	99.52	100.00	99.52	99.76	90.00	88.10	89.29	87.86	88.81	90.24	71.90	71.90	71.90	75.00	75.48	65.95	67.86	67.62	69.05	68.81	69.05	69.21	68.74	70.71	70.88	71.36	71.36	71.12
16: S.saprophyticus-UTI-035	74.05	74.29	74.29	74.52	79.76	79.29	79.05	79.05	86.67	86.67	89.29	89.29	99.52	99.52	99.52	100.00	99.76	90.00	88.10	89.76	87.86	88.81	90.24	71.90	71.90	71.90	75.00	75.48	66.43	67.86	67.62	69.52	69.29	69.52	69.69	69.21	71.19	71.36	71.84	71.84	71.60
17: S.saprophyticus-FDAARGOS_137	73.81	74.05	74.05	74.29	79.52	79.05	78.81	78.81	86.43	86.43	89.05	89.05	99.29	99.29	99.76	99.76	100.00	89.76	87.86	89.52	87.62	88.57	90.00	71.67	71.67	71.67	74.76	75.24	66.19	67.62	67.38	69.29	69.05	69.29	69.45	68.97	70.95	71.12	71.60	71.60	71.36
18: S.cohnii-ATCC29974	75.24	75.24	75.48	75.71	80.48	79.76	79.52	79.52	88.33	88.33	89.52	88.10	90.48	90.00	90.00	90.00	89.76	100.00	89.76	90.48	90.00	91.19	90.71	71.90	72.38	72.38	73.33	74.05	65.71	67.62	67.38	69.76	70.00	69.76	69.69	69.21	69.29	69.93	70.41	70.41	70.17
19: S.saprophyticus-P18-2	74.29	74.52	74.52	74.76	79.76	80.24	79.76	79.76	87.62	87.62	88.33	90.48	88.57	88.10	88.10	88.10	87.86	89.76	100.00	90.24	89.29	91.19	90.95	71.90	71.67	71.67	74.76	75.24	66.67	66.19	65.95	67.86	67.62	67.86	69.69	69.21	70.95	71.60	71.60	71.60	71.36
20: S.saprophyticu-FDAARGOS_336	73.81	74.05	74.05	74.29	78.81	77.62	77.14	77.14	85.95	85.95	88.10	89.05	89.76	89.29	89.29	89.76	89.52	90.48	90.24	100.00	91.90	91.19	90.71	71.43	71.43	71.43	75.00	74.76	66.67	65.95	65.71	70.95	71.19	70.95	69.21	68.97	70.24	70.41	70.88	70.88	70.64
21: S.xylosus-ATCC29971	73.10	73.33	73.33	73.57	80.24	78.81	78.10	78.10	85.71	85.71	89.76	90.24	88.33	87.86	87.86	87.86	87.62	90.00	89.29	91.90	100.00	91.43	88.10	71.19	70.71	70.71	72.86	73.10	65.95	65.00	64.76	70.48	70.24	70.48	67.54	67.30	70.24	69.93	70.41	70.41	70.17
22: S.saprophyticus-M17-3	76.19	76.19	76.43	76.67	80.95	79.76	79.29	79.29	86.67	86.67	90.00	90.71	89.29	88.81	88.81	88.81	88.57	91.19	91.19	91.19	91.43	100.00	91.67	70.24	70.24	70.24	73.33	73.57	65.48	67.38	67.14	70.24	70.48	70.24	68.74	68.50	70.00	69.69	70.17	70.17	69.93
23: S.succinus-14BME20	78.33	78.33	78.57	78.81	81.19	80.48	79.52	79.52	86.90	86.90	88.57	89.05	90.71	90.24	90.24	90.24	90.00	90.71	90.95	90.71	88.10	91.67	100.00	73.81	73.81	73.81	75.71	75.71	66.90	68.57	68.10	70.48	70.24	70.48	71.60	71.12	71.67	71.84	72.32	72.32	72.08
24: S.aureus-MRSA252	69.29	69.05	69.52	69.52	67.14	66.43	65.95	65.95	71.90	71.90	70.95	70.24	71.90	71.90	71.90	71.90	71.67	71.90	71.90	71.43	71.19	70.24	73.81	100.00	99.05	98.57	79.05	79.05	66.90	68.81	68.33	68.57	68.81	68.81	70.17	69.69	70.00	70.41	70.64	70.64	70.41
25: S.aureus-N315	69.52	69.29	69.76	69.76	67.14	66.43	65.95	65.95	71.67	71.67	70.95	70.24	71.90	71.90	71.90	71.90	71.67	72.38	71.67	71.43	70.71	70.24	73.81	99.05	100.00	99.52	79.05	79.05	67.38	68.81	68.33	68.81	69.05	69.05	69.93	69.45	70.24	70.64	71.36	71.36	71.12
26: S.aureus-M92	69.52	69.29	69.76	69.76	66.90	66.19	65.71	65.71	71.43	71.43	70.71	70.24	71.90	71.90	71.90	71.90	71.67	72.38	71.67	71.43	70.71	70.24	73.81	98.57	99.52	100.00	78.81	78.81	67.62	68.81	68.33	68.57	68.81	68.81	69.93	69.45	70.24	70.64	71.36	71.36	71.12
27: S.aureus-JS395	70.71	70.71	70.95	70.71	70.24	69.52	69.52	69.52	74.05	74.05	74.29	74.29	75.00	75.00	75.00	75.00	74.76	73.33	74.76	75.00	72.86	73.33	75.71	79.05	79.05	78.81	100.00	98.33	64.76	69.52	69.52	69.76	69.76	69.52	71.12	70.88	72.14	72.32	72.32	72.32	72.55
28: S.cohnii-FDAARGOS_744	70.48	70.48	70.71	70.48	70.48	70.24	70.24	70.24	74.29	74.29	74.76	74.76	75.48	75.48	75.48	75.48	75.24	74.05	75.24	74.76	73.10	73.57	75.71	79.05	79.05	78.81	98.33	100.00	64.76	69.52	69.52	70.24	70.24	70.00	71.84	71.60	72.62	72.79	72.79	72.79	73.03
29: S.anthracis-fosB2	64.05	64.29	64.29	64.52	66.19	66.67	65.71	66.19	65.48	65.48	65.00	66.90	66.43	66.19	65.95	66.43	66.19	65.71	66.67	66.67	65.95	65.48	66.90	66.90	67.38	67.62	64.76	64.76	100.00	63.10	62.38	70.95	70.95	71.19	63.96	63.96	65.48	65.87	65.63	65.63	65.87
30: S.epidermidis-ATCC12228	67.62	67.14	67.38	67.62	67.86	67.14	66.67	66.90	66.67	66.67	67.14	66.43	67.86	68.33	67.86	67.86	67.62	67.62	66.19	65.95	65.00	67.38	68.57	68.81	68.81	68.81	69.52	69.52	63.10	100.00	99.07	71.43	71.67	71.43	69.63	69.39	69.23	69.91	69.91	69.91	69.91
31: S.epidermidis-RP62A	67.38	66.90	67.14	67.38	67.62	66.90	66.43	66.67	66.43	66.43	66.90	66.19	67.62	68.10	67.62	67.62	67.38	67.38	65.95	65.71	64.76	67.14	68.10	68.33	68.33	68.33	69.52	69.52	62.38	99.07	100.00	70.95	71.19	70.95	68.93	68.69	68.53	69.19	69.19	69.19	69.19
32: S.lugdunensis-APC3758	70.71	70.71	71.19	71.43	70.00	71.19	70.00	70.48	67.62	67.62	69.29	69.76	69.52	69.29	69.05	69.52	69.29	69.76	67.86	70.95	70.48	70.24	70.48	68.57	68.81	68.57	69.76	70.24	70.95	71.43	70.95	100.00	99.52	99.76	72.08	72.08	73.10	72.55	72.79	72.79	73.03
33: S.lugdunensis-HKU09-01	70.95	70.95	71.43	71.67	70.24	70.95	70.24	70.71	67.86	67.86	69.52	69.52	69.29	69.05	68.81	69.29	69.05	70.00	67.62	71.19	70.24	70.48	70.24	68.81	69.05	68.81	69.76	70.24	70.95	71.67	71.19	99.52	100.00	99.76	72.55	72.55	73.57	73.03	73.27	73.27	73.51
34: S.lugdunensis-JICS135	70.71	70.71	71.19	71.43	70.00	71.19	70.00	70.48	67.62	67.62	69.29	69.76	69.52	69.29	69.05	69.52	69.29	69.76	67.86	70.95	70.48	70.24	70.48	68.81	69.05	68.81	69.52	70.00	71.19	71.43	70.95	99.76	99.76	100.00	72.32	72.32	73.33	72.79	73.03	73.03	73.27
35: S.caprae-26D	72.55	72.79	73.03	73.27	67.54	68.26	67.54	68.02	65.67	65.67	67.78	67.06	69.21	69.45	69.21	69.69	69.45	69.69	69.69	69.21	67.54	68.74	71.60	70.17	69.93	69.93	71.12	71.84	63.96	69.63	68.93	72.08	72.55	72.32	100.00	98.86	84.02	85.34	85.34	85.34	85.58
36: S.caprae-NCTC12196	71.60	71.84	72.08	72.32	67.54	68.26	67.54	68.02	65.21	65.21	67.78	66.59	68.74	68.97	68.74	69.21	68.97	69.21	69.21	68.97	67.30	68.50	71.12	69.69	69.45	69.45	70.88	71.60	63.96	69.39	68.69	72.08	72.55	72.32	98.86	100.00	83.33	84.63	84.63	84.63	84.87
37: S.capitis-SK14	71.90	71.67	72.14	72.38	69.29	69.05	68.57	69.05	66.30	66.67	70.71	69.52	70.71	70.95	70.71	71.19	70.95	69.29	70.95	70.24	70.24	70.00	71.67	70.00	70.24	70.24	72.14	72.62	65.48	69.23	68.53	73.10	73.57	73.33	84.02	83.33	100.00	98.11	99.05	99.05	99.29
38: S.capitis-C2	72.08	71.84	72.32	72.55	69.21	68.97	68.50	68.97	68.74	68.74	70.88	69.69	70.88	71.12	70.88	71.36	71.12	69.93	71.60	70.41	69.93	69.69	71.84	70.41	70.64	70.64	72.32	72.79	65.87	69.91	69.19	72.55	73.03	72.79	85.34	84.63	98.11	100.00	98.58	98.58	98.82
39: S.capitis-BN2	72.32	72.08	72.55	72.79	69.45	69.21	68.74	69.21	69.45	69.45	71.36	70.17	71.36	71.60	71.36	71.84	71.60	70.41	71.60	70.88	70.41	70.17	72.32	70.64	71.36	71.36	72.32	72.79	65.63	69.91	69.19	72.79	73.27	73.03	85.34	84.63	99.05	98.58	100.00	100.00	99.76
40: S.capitis-C46-2	72.32	72.08	72.55	72.79	69.45	69.21	68.74	69.21	69.45	69.45	71.36	70.17	71.36	71.60	71.36	71.84	71.60	70.41	71.60	70.88	70.41	70.17	72.32	70.64	71.36	71.36	72.32	72.79	65.63	69.91	69.19	72.79	73.27	73.03	85.34	84.63	99.05	98.58	100.00	100.00	99.76
41: S.capitis-C1	72.32	72.08	72.55	72.79	69.69	69.45	68.97	69.45	69.21	69.21	71.12	69.93	71.12	71.36	71.12	71.60	71.36	70.17	71.36	70.64	70.17	69.93	72.08	70.41	71.12	71.12	72.55	73.03	65.87	69.91	69.19	73.03	73.51	73.27	85.58	84.87	99.29	98.82	99.76	99.76	100.00
**strain/isolate ID**	1	2	3	4	5	6	7	8	9	10	11	12	13	14	15	16	17	18	19	20	21	22	23	24	25	26	27	28	29	30	31	32	33	34	35	36	37	38	39	40	41

Strain/isolate ID shown at the bottom corresponds to those listed on the leftmost column. Clusters classified by staphylococcal species and subspecies are indicated on the top, and identities within the same clusters/subclusters are shown by colored shading/square.
Same identities are shown in the right upper and left lower portions for individual combinations of strains. *S. anthracis-fosB2* (no.29) (GenBank accession no.NC_012581) was added as a reference belonging to one of the three major *fosB* phylogenetic groups (FosB1) that is distinct from *Staphylococcus* group (FosB-S).
