# Supplementary material for: Prevalence and Antimicrobial Resistance of Staphylococcus aureus and Coagulase-Negative Staphylococcus/Mammaliicoccus from Retail Ground Meat: Identification of Broad Genetic Diversity in Fosfomycin Resistance Gene fosB
Source: Pathogens. 2022 Apr 14;11(4):469. doi: 10.3390/pathogens11040469 (PMC9031665; doi:10.3390/pathogens11040469)
Supplement: Supplementary file 1 [file pathogens-11-00469-s001.zip › Supplementary Tables S2-S5.pdf]

Table S2 Primers to detect 6-TG biosynthesis cluster genes by PCR

Target gene	primer	Nucleotide sequence	Product size
tgsB	tgsB-1	ATGGAAAGAGAAAAAGTTGC	698 bp
	tgsB-2	AAAAGACTCCTTAATATTGG	
tgsC	tgsC-1	GATAAATTAGACATATGTGA	560 bp
	tgsC-2	CTATATTAGAATTAAAACCAAATG	
tgsD	tgsD-1	GGTAGTGGCATTGCTAAATTTG	568 bp
	tgsD-2	CATATTTGTTGCAAATGATACTC	

Table S3 Primers used to detect *fosB* by PCR for different *Staphylococus* species and *Enterococcus*

Bacterial species	Primer	Nucleotide sequence	Product size
S. aureus	SA-fosB-F1	CATATATGCTTTTCAGTCAG	332 bp
	SA-fosB-R1	TGTAATTCTAGCTTATGACC	
S. capitis	Scapi-fosB-NF1	TCGTAAATCAAGTAACGAAG	515 bp
	Scapi-fosB-RF1	GAAGTTAAATAGTATTTTAGAG	
S. caprae	Scaprae-fosB-F1	CATGTTTGCTTTTCTGTTAG	328 bp
	Scaprae-fosB-R1	ATTCTAATTTATGCCCATC	
S. epidermidis	Sepi-fosB-F1	GTCATACTTGGATTGCACTG	219 bp
	Sepi-fosB-F1	ATTCAATTTTATGCCCATCC	
S. haemolyticus	Shaemo-fosB--F1	GATATTTTAGGGGCTGACAT	293 bp
	Shaemo-fosB--R1	AAACTTCCTGTATGCAATTC	
S. lugdunensis	Slugd-fosB-F1	TCTGTAGCTGATTTAGCGAC	309 bp
	Slugd-fosB-R1	TTTATGACCATCTGGATCTG	
S. pasteuri	Spaste-fosB-NF1	ATGATTCAATCCGTAAATCAC	420 bp
	Spaste-fosB-NR1	CTATTCATAAAATTTTATATGAG	
S. saprophyticus	C571-FB-5P1	ATGATTCAATCTATAAATCAC	425 bp (+C571-FB-3P1)
	C571-FB-3P1	CCTACTTATATATAAAATTTCATATG	
	M173-FB3	GATTCAATCTATAAACCACG	201 bp (+M173-FB4)
	M173-FB4	GCCATATGTGTATACGAGTA	
S.warneri	Swarn-fosB-F2	TAAATAGGAGTAATTATATG	600 bp
	Swarn-fosB-R2	TATAAACTATTATTTACCTA	
E. faecium	Efm-fosB-F1	TACAGAGATATTTTAGGGGC	309bp
	Efm-fosB-R1	TCTATCTTCTAAACTTCCTG	

Table S4 Primers used to amplify whole *fosB* ORF to determine its nucleotide sequence

Staphylococcal species	Primer	Nucleotide sequence	Product size
S.saprophyticus	S.sap-fosB-N4	(+) TCTATTTTAGGAGTTTTTATATG	1.5 kb (+M173-R1)
	M173-R1	(-) ATGCCATATGTGTATACGAG	
	M173-FB1	(+) CA CAATAAGCGA GCTGTATC	1.1 kb (+M173-FB2)
	M173-FB2	(-) CTCTGTACTGCATCGAGAAATG	
	M173-FB7	(-)TCGCTCTGTGTTGCATCGAT	1 kb (+M173-FB1)
	C571-FB-UPF1	(+) ATG GAGAAAAAAT TGAAGTGA	1.5 kb (+C571-FB-DW1)
	C571-FB-DW1	(-) CATTGTAAAAAACTTCCCCA	
	P182-5'-F1	(+) TGGGGTGAATTAACATATGAG	1 kb (+P182+5'-R1)
	P182-5'-R1	(-) CATCTTTATAGAAAGCAATAG	
S.warneri, S. pasteuri, S. lugdunensis	Swarn-fosB-F2	(+) TAAATAGGAGTAATTATATG	600 bp (+Swarn-fosB-R2)
	Swarn-fosB-R2	(-) TATAAACTATTATTTACCTA	
S.capitis	Scapi-fosB-NF1	(+) TCGTAAATCAAGTAACGAAG	515 bp (+Scapi-fosB-nR1)
	Scapi-fosB-NR1	(-) GAAGTTAAATAGTATTTTAGAG	

Table S5 Genbank accession numbers of *fosB* gene identified in this study

Isolate ID	Specimen (ground meat)	Species	GenBank accession no.
C47-3	chicken	*S. lugdunensis*	OL964368
C34-2	chicken	*S. pasteuri*	OL964369
C1	chicken	*S. capitis*	OL964370
C2	chicken	*S. capitis*	OL964371
C46-2	chicken	*S. capitis*	OL964372
C6	chicken	*S. warneri*	OL964373
C33-1	chicken	*S. warneri*	OL964374
C39-2	chicken	*S. saprophyticus*	OL964375
P6-1	pork	*S. saprophyticus*	OL964376
M25-2	pork and beef	*S. saprophyticus*	OL964377
C55-2	chicken	*S. saprophyticus*	OL964378
C60-1	chicken	*S. saprophyticus*	OL964379
C44-2	chicken	*S. saprophyticus*	OL964380
C57-1	chicken	*S. saprophyticus*	OL964381
P18-2	pork	*S. saprophyticus*	OL964382
M17-3	pork and beef	*S. saprophyticus*	OL964383
