# Supplementary material for: Prevalence and Antimicrobial Resistance of Staphylococcus aureus and Coagulase-Negative Staphylococcus/Mammaliicoccus from Retail Ground Meat: Identification of Broad Genetic Diversity in Fosfomycin Resistance Gene fosB
Source: Pathogens. 2022 Apr 14;11(4):469. doi: 10.3390/pathogens11040469 (PMC9031665; doi:10.3390/pathogens11040469)
Supplement: Supplementary file 1 [file pathogens-11-00469-s001.zip › Supplementary Figure S1-MIC.pptx]

## Slide 1
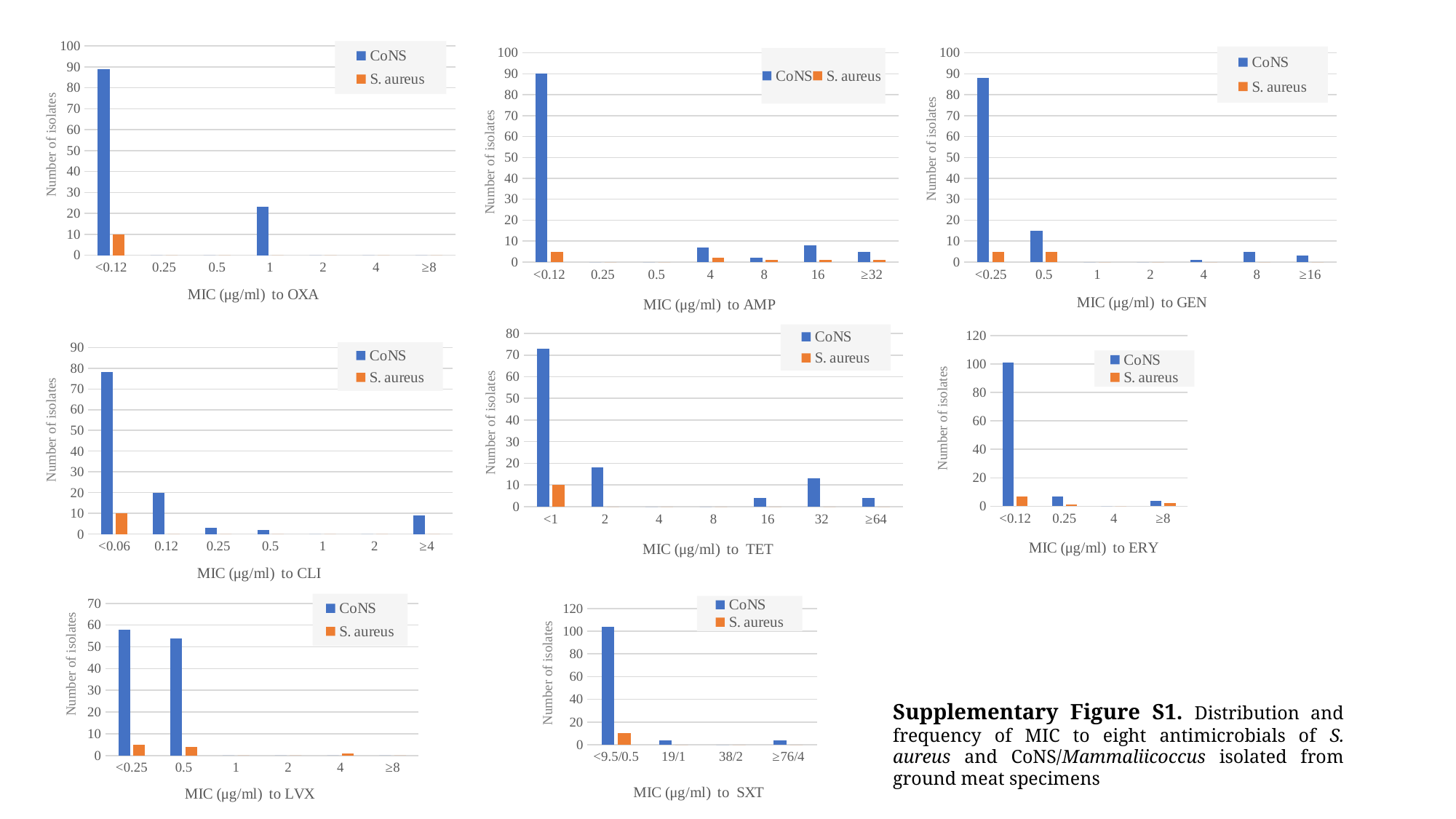

### Chart: MIC (μg/ml) to AMP
| Category | CoNS | S. aureus |
|---|---|---|
| <0.12 | 90.0 | 5.0 |
| 0.25 | 0.0 | 0.0 |
| 0.5 | 0.0 | 0.0 |
| 4 | 7.0 | 2.0 |
| 8 | 2.0 | 1.0 |
| 16 | 8.0 | 1.0 |
| ≥32 | 5.0 | 1.0 |
### Chart: MIC (μg/ml) to OXA
| Category | CoNS | S. aureus |
|---|---|---|
| <0.12 | 89.0 | 10.0 |
| 0.25 | 0.0 | 0.0 |
| 0.5 | 0.0 | 0.0 |
| 1 | 23.0 | 0.0 |
| 2 | 0.0 | 0.0 |
| 4 | 0.0 | 0.0 |
| ≥8 | 0.0 | 0.0 |
### Chart: MIC (μg/ml) to GEN
| Category | CoNS | S. aureus |
|---|---|---|
| <0.25 | 88.0 | 5.0 |
| 0.5 | 15.0 | 5.0 |
| 1 | 0.0 | 0.0 |
| 2 | 0.0 | 0.0 |
| 4 | 1.0 | 0.0 |
| 8 | 5.0 | 0.0 |
| ≥16 | 3.0 | 0.0 |Number of isolates
Number of isolates
Number of isolates
### Chart: MIC (μg/ml) to TET
| Category | CoNS | S. aureus |
|---|---|---|
| <1 | 73.0 | 10.0 |
| 2 | 18.0 | 0.0 |
| 4 | 0.0 | 0.0 |
| 8 | 0.0 | 0.0 |
| 16 | 4.0 | 0.0 |
| 32 | 13.0 | 0.0 |
| ≥64 | 4.0 | 0.0 |
### Chart: MIC (μg/ml) to CLI
| Category | CoNS | S. aureus |
|---|---|---|
| <0.06 | 78.0 | 10.0 |
| 0.12 | 20.0 | 0.0 |
| 0.25 | 3.0 | 0.0 |
| 0.5 | 2.0 | 0.0 |
| 1 | 0.0 | 0.0 |
| 2 | 0.0 | 0.0 |
| ≥4 | 9.0 | 0.0 |
### Chart: MIC (μg/ml) to ERY
| Category | CoNS | S. aureus |
|---|---|---|
| <0.12 | 101.0 | 7.0 |
| 0.25 | 7.0 | 1.0 |
| 4 | 0.0 | 0.0 |
| ≥8 | 4.0 | 2.0 |Number of isolates
Number of isolates
Number of isolates
### Chart: MIC (μg/ml) to LVX
| Category | CoNS | S. aureus |
|---|---|---|
| <0.25 | 58.0 | 5.0 |
| 0.5 | 54.0 | 4.0 |
| 1 | 0.0 | 0.0 |
| 2 | 0.0 | 0.0 |
| 4 | 0.0 | 1.0 |
| ≥8 | 0.0 | 0.0 |
### Chart: MIC (μg/ml) to SXT
| Category | CoNS | S. aureus |
|---|---|---|
| <9.5/0.5 | 104.0 | 10.0 |
| 19/1 | 4.0 | 0.0 |
| 38/2 | 0.0 | 0.0 |
| ≥76/4 | 4.0 | 0.0 |Number of isolates
Number of isolates
Supplementary Figure S1. Distribution and frequency of MIC to eight antimicrobials of S. aureus and CoNS/Mammaliicoccus isolated from ground meat specimens
